# A Review of Research on Technology-Supported Language Learning and 21st Century Skills

**DOI:** 10.3389/fpsyg.2022.897689

**Published:** 2022-07-07

**Authors:** Rustam Shadiev, Xun Wang

**Affiliations:** School of Education Science, Nanjing Normal University, Nanjing, China

**Keywords:** language learning, 21st century skills, technology, review, development

## Abstract

Modern society needs people to be equipped with 21st century skills (e.g., critical thinking, creativity, communication, digital literacy, or collaboration skills). For this reason, teaching and learning nowadays should promote not only students' knowledge acquisition in various learning contexts but also their 21st century skills, and language learning context is no exception. This study reviewed research on technology-supported language learning and 21st century skills. The reason is that earlier studies reviewed only articles related to language learning supported by technology and mostly focused on languages, language skills and technologies used. That is to say, 21st century skills were not considered in earlier review studies. The present study selected and reviewed 34 articles published between 2011 and 2022 (February) and focused on the following dimensions: (1) research focus such as language skills and 21st century skills; (2) theoretical foundations; (3) technologies; (4) learning activities; (5) methodology; and (6) findings. The present research found that reviewed studies had focused most frequently on such language skills as speaking and writing and on such 21st century skills as communication and collaboration. The social constructivism theory was often used by scholars to base their studies on. Facebook, Google Docs, and Moodle were popular technologies in reviewed studies to facilitate language and 21st century skills. Scholars in reviewed studies reported that technology-supported language learning activities provided learners with good learning experiences and enhanced their learning motivation, engagement, and confidence. However, some challenges that learners faced during learning activities were also reported. Based on the results of the review, this study made several recommendations for stakeholders such as educators and researchers in the field.

## Introduction

It is important that our students not only acquire new knowledge when they learn, but also develop skills, such as problem-solving, social cooperation, creativity, and so on, in order to apply newly learned knowledge to the real world. Such knowledge and skills will help them adapt to modern society and will enhance their competitiveness (Shadiev et al., 2022a,b). Many countries have put forward the 21st century skills framework to carry out education reform (Lin et al., 2020), and one of them was proposed by the Partnership for 21st Century (P21). The P21 (Partnership for 21st Century Skills, 2008) provided a detailed conceptual framework and listed three types of skills: (1) learning and innovation skills (critical thinking and problem solving, creativity and innovation, and communication and collaboration), (2) digital literacy (information literacy, media literacy, and information and communication technologies (ICT) literacy), and (3) career and life skills (flexibility and adaptability, initiative and self-direction, social and cross-cultural interaction, productivity and accountability, and leadership and responsibility). The essence of these skills is that they are key skills that learners will need for their social and professional life in the future. These skills also emphasize the ability of learners to use and transfer knowledge and solve problems in complex situations, so they can achieve deep levels of individual learning as well as lifelong learning (Shadiev et al., 2022a).

Developing students' 21st century skills needs to be implemented in all disciplines, and foreign language learning is no exception (Shadiev et al., In Press). This matter has been addressed in the documents related to Asia Pacific Economic Cooperation (2004). Furthermore, researchers have carried out related studies, and pointed out the advantages of technology in developing both language skills and 21st century skills (Shadiev et al., In Press). For example, Suzanne (2014) pointed out that when developing learners' reading skills, they deepened the learners' understanding of reading content, and also developed critical thinking skills. García-Sánchez and Burbules (2016) have found that students' skills such as problem solving, collaboration, listening and speaking improved after they completed online collaborative tasks. Srebnaja and Stavicka (2018) also pointed out that, in language learning projects supported by WebQuest, students' creativity, collaboration, and speaking skills have been developed. In the study by Chiang (2020), the digital storytelling activity was designed which promoted language learners' writing skills as well as their digital literacy skills.

A theoretical foundation to support technology supported language learning and development of 21st century skills can be built on various theories. The most relevant can be considered as second language acquisition theory, socio-cultural theory, and constructivism theory. For example, second language acquisition theory states that language acquisition is a process of input, absorption, and output. Language acquisition is acquired through exposure to contexts, understanding discourse, and then using language in natural communicative contexts (Krashen, 1985). According to socio-cultural theory, learning is a social phenomenon; it emphasizes the social nature of learning and argues that the development of learners' abilities arises from interpersonal interactions (Lantolf, 2000). Constructivism theory suggests that learning is a process in which a learner actively constructs meaning. That is, learners generate meaning and construct understanding based on prior knowledge and experience, often in the context of socio-cultural interactions. Constructivism theory emphasizes the social and contextual nature of learning (Vygotsky, 1978). Over the years, scholars have created technology-supported learning environments for language learning and 21st century skills development based on these theories. Such environments provide students with authentic learning materials, support social interaction, and facilitate their creative expression and construction of meaning actively using the target language.

Some related review studies already exist in the field. For example, Shadiev and Yang (2020) reviewed 398 articles related to technology-assisted language learning published in 10 Social Science Citation Index (SSCI) journals. The dimensions analyzed in their study included target language, language skills, technologies, and research findings. Shadiev and Yang (2020) found that the most commonly used language was English, followed by Chinese. The most targeted language skills were writing, speaking, and vocabulary acquisition. Digital games and online videos were the most commonly used technologies in these reviewed studies. In addition, most of the reviewed studies reported positive impacts of technology applications on language learning. Zhang and Zou (2020) reviewed 57 articles on technology applications for language learning that were published between 2016 and 2019 in 10 SSCI journals. The types of technology, the purpose of technology use, and the effectiveness of the technologies were reviewed by the authors. Zhang and Zou (2020) found that mobile learning, multimedia learning and socialization, voice to text recognition, text to speech recognition, and digital game-based learning were the most frequently investigated types of technology in the literature. The purposes for their use mainly covered four areas, including promoting practice, providing teaching content, promoting interaction, and reconstructing teaching methods. Scholars have claimed that technologies have positive effects on language learning. Goksu et al. (2020) reviewed 310 articles in 10 journals in the field of technology-assisted language learning. In addition, they evaluated a metadata set of 469 articles in the Web of Science database through bibliometric mapping. The review focused on the types, purposes, and effectiveness of the latest technologies on language learning. Goksu et al. (2020) found that most studies used quantitative research methods and were carried out with participants at higher academic levels. In addition, most studies focused on language skills as well as learning motivation and learner perceptions. Shadiev et al. (2017) studied 37 articles published in the top 10 SSCI journals related to educational technology from 2007 to 2016 (March). Scholars took mobile language learning in a real environment as the research object and summarized the results from four perspectives: journal publishing trends, language learning, research focus, and research methods. The results showed that the journal publishing trend was increasing. The most common research focus was cognition and language learner proficiency. The results also showed that mobile technology was positively perceived and accepted by students in most of these studies, and the technology was also found to have a positive impact on the students' language skills.

By exploring these review studies, the present review research found that 21st century skills were not considered in these earlier studies at all because scholars mainly focused on language skills. Therefore, several important aspects (e.g., theoretical foundations used to support the studies, methodology, and types of learning activities that promote language skills and 21st century skills) were ignored. These aspects are important for stakeholders in the design and implementation of language teaching and learning for 21st century skills development. In order to fill this gap in the literature, the present study was carried out, and the following research questions were addressed:

1. What language skills and 21st century skills did the researchers focus on in the reviewed studies?
2. What theories were used as a foundation in reviewed studies?
3. What technologies were used to promote language skills and 21st century skills?
4. What learning activities were used in the reviewed studies?
5. What were the methodological characteristics of the reviewed studies?
6. What research findings were obtained in the reviewed studies?

## Research Method

**Figure 1:**
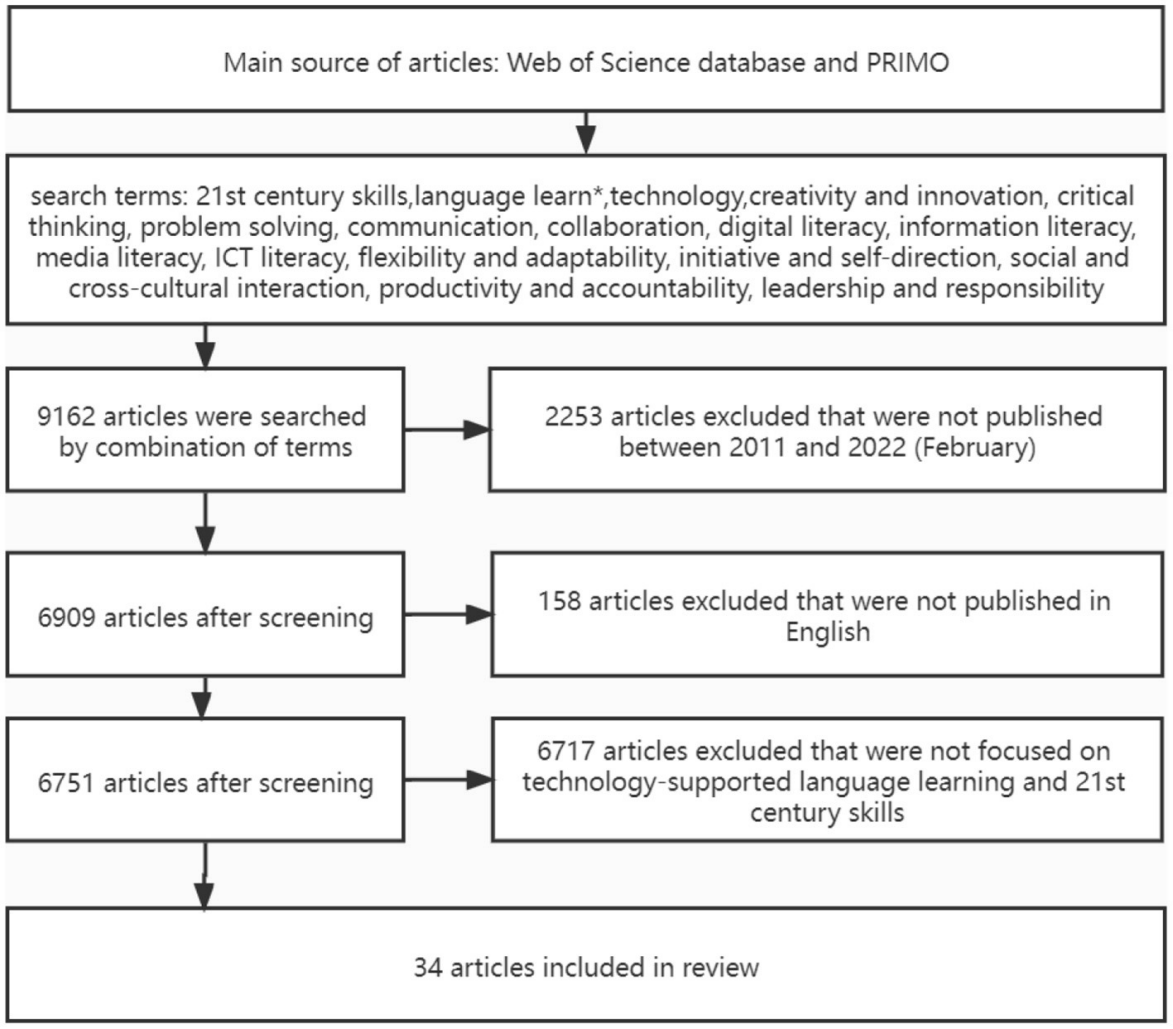
The search and selection process.

The present study is a systematic review. The study used preferred reporting items for systematic reviews and meta-analyses (PRISMA) for the electronic search. PRISMA is considered as a set of programs that facilitates researchers to prepare and report various systematic evaluations and meta-analyses (Moher et al., 2009). According to scholars, PRISMA has been widely and successfully applied in educational research. In addition to PRISMA, this review followed the general guidelines for searching and selecting research articles proposed by Avgousti (2018), Shadiev and Yang (2020), and Shadiev and Yu (In Press). The search and selection processes are shown in Figure 1. Articles were found through a search on the Web of Science database and Peer-Reviewed Instructional Materials Online Database (PRIMO). According to Kukulska-Hulme and Viberg (2018), PRIMO is a search tool and it contains several databases such as ERIC and Scopus. For this reason, PRIMO features a very comprehensive collection of full-text articles and bibliographic records, and it has been used by many researchers in their systematic reviews and meta-analyses to find relevant literature.

Based on general recommendations from previous review studies (Guan, 2014; Duman et al., 2015), this review used keywords such as 21st century skills, language learn^*^, and technology. 21st century skills were also included to widen the search results (e.g., creativity and innovation, critical thinking, problem solving, communication, collaboration, digital literacy, information literacy, media literacy, ICT literacy, flexibility and adaptability, initiative and self-direction, social and cross-cultural interaction, productivity and accountability, leadership and responsibility). This review used these terms in different combinations to search articles.

A total of 9,162 articles were found from the search. This review narrowed down the selection of research articles based on the following criteria (see Figure 1): articles that were (1) published during 2011–2022 (February); (2) published in English; and (3) focused on technology-supported language learning and 21st century skills. Two researchers screened each article individually and excluded articles from the study that did not focus on technology-supported language learning and 21st century skills. The researchers discussed any discrepancies in their selection results until an agreement was reached. At the end of the selection process, 34 empirical studies were chosen for the review.

**Figure 2:**
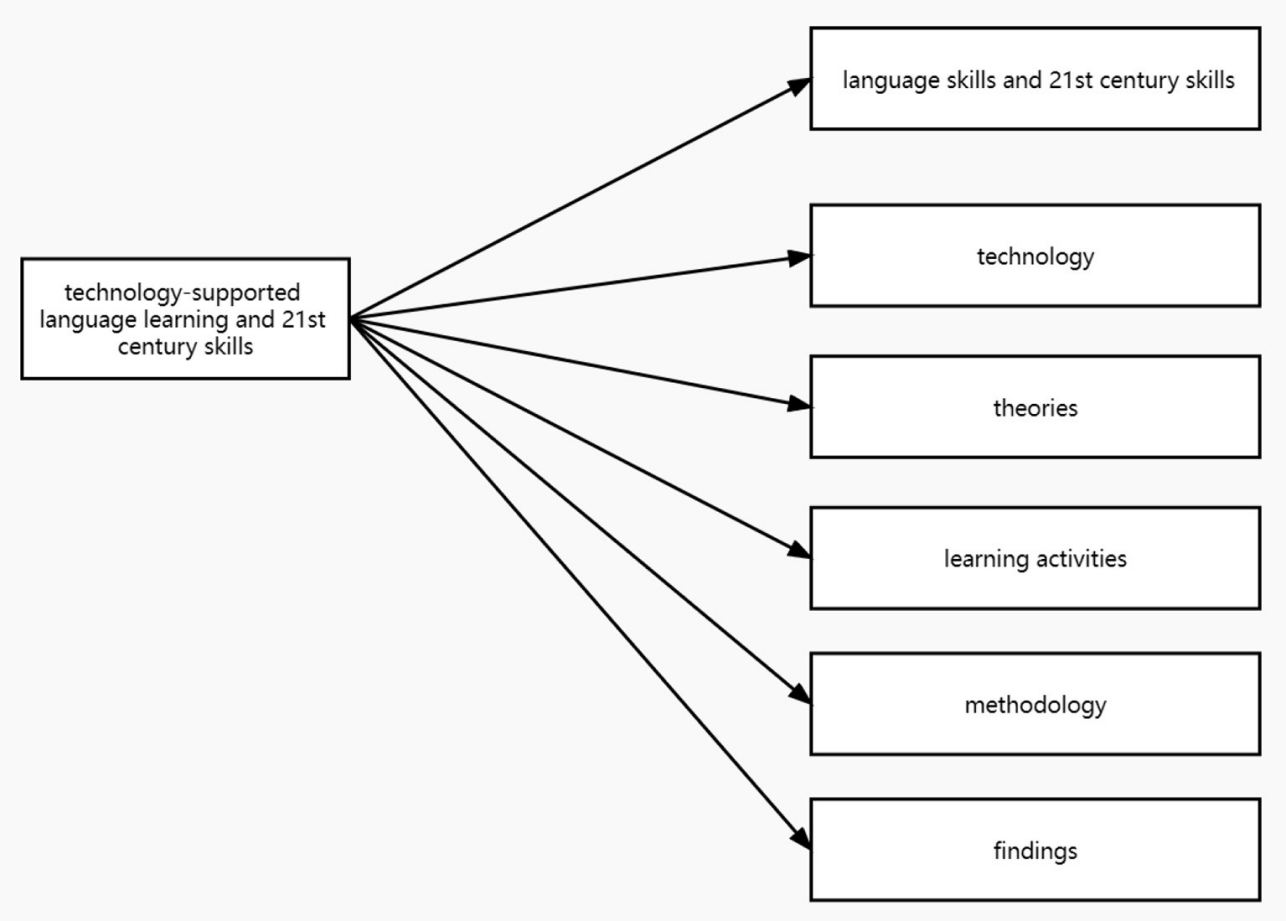
Analytical framework.

This review proposed an analytical framework (see Figure 2) to answer the research questions of the study and to better understand the research design of the reviewed studies and findings. This review also used this framework to help us better review articles and regarded it as the basis for coding the content of reviewed studies. This review used the open coding method to carry out content analysis (Creswell, 2002) which can enable us to segment research content and to form categories relevant to the phenomenon of interest. The analytical dimensions included the following (see Figure 2): (1) language skills and 21st century skills—skills related to language learning and 21st century skills, (2) technology—the tools and devices participants used for language learning, (3) learning activities supported by technology to cultivate 21st century skills and language skills, (4) theoretical foundation—theories, models or hypothesis involved in research, (5) methodology—participants' academic level and major, research duration, sample size, data collection tools, and research design, and (6) findings—results reported in research.

Two researchers were involved in the coding process. They read articles and coded content according to the above coding scheme. After that, they categorized codes into categories and identified attributes for each category. If there were any differences in coding, the researchers re-examined an article to resolve differences, and then finally completed the coding phase. Interrater reliability was measured using Cohen's kappa coefficient and the result was high (k = 0.886).

## Results

**Figure 3:**
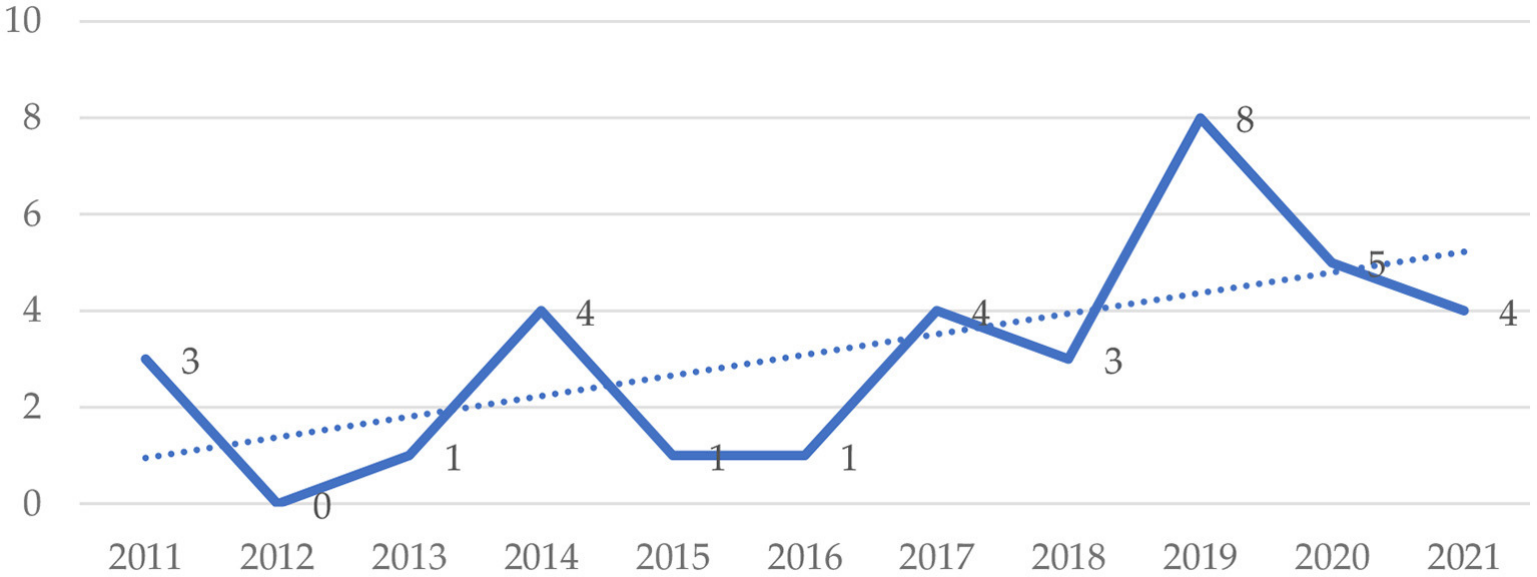
Distribution of research year.

**Figure 4:**
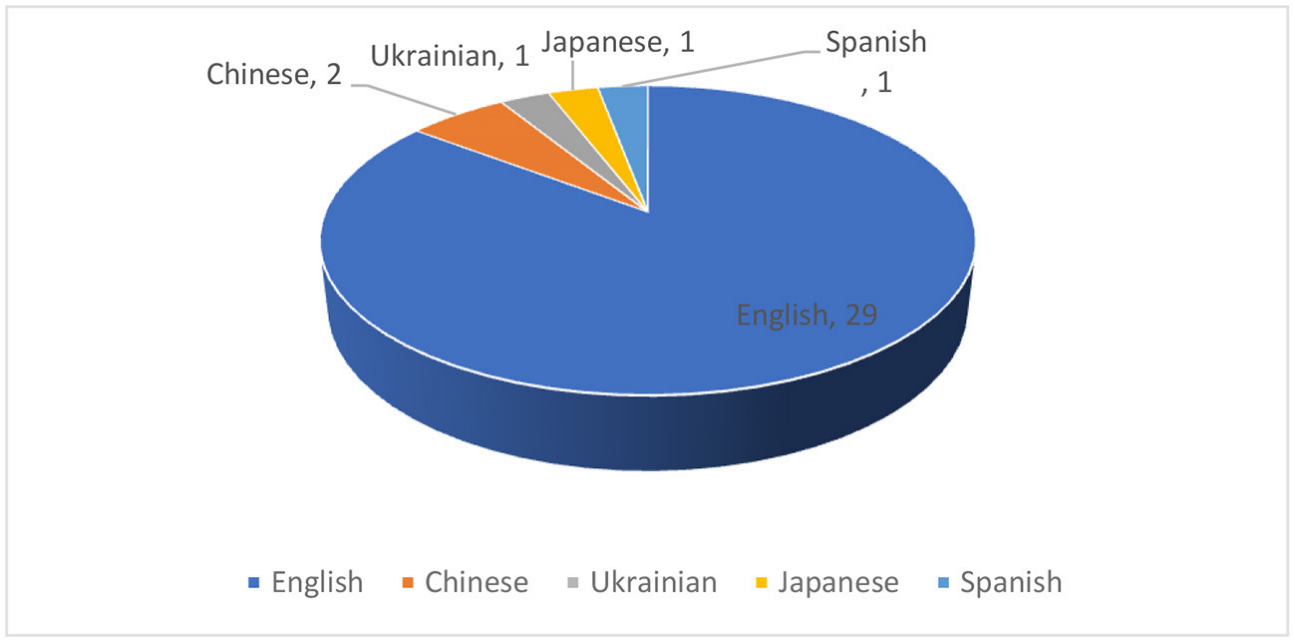
Distribution of languages.

**Figure 5:**
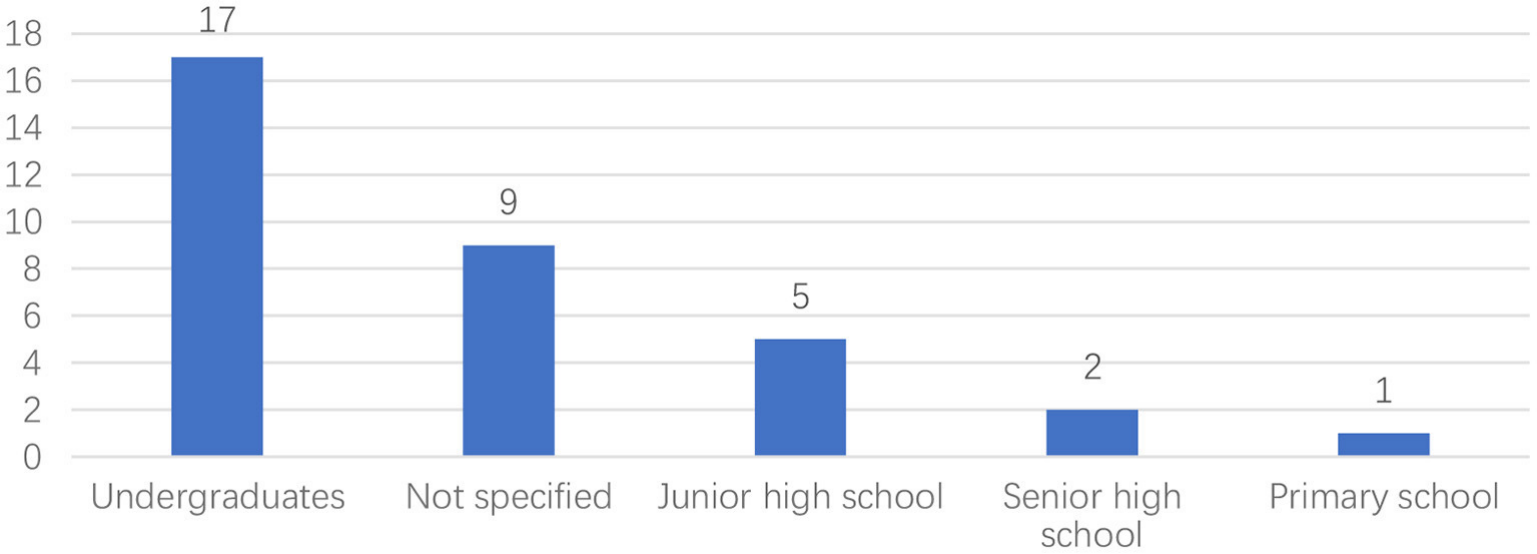
Distribution of educational level.

**Figure 6:**
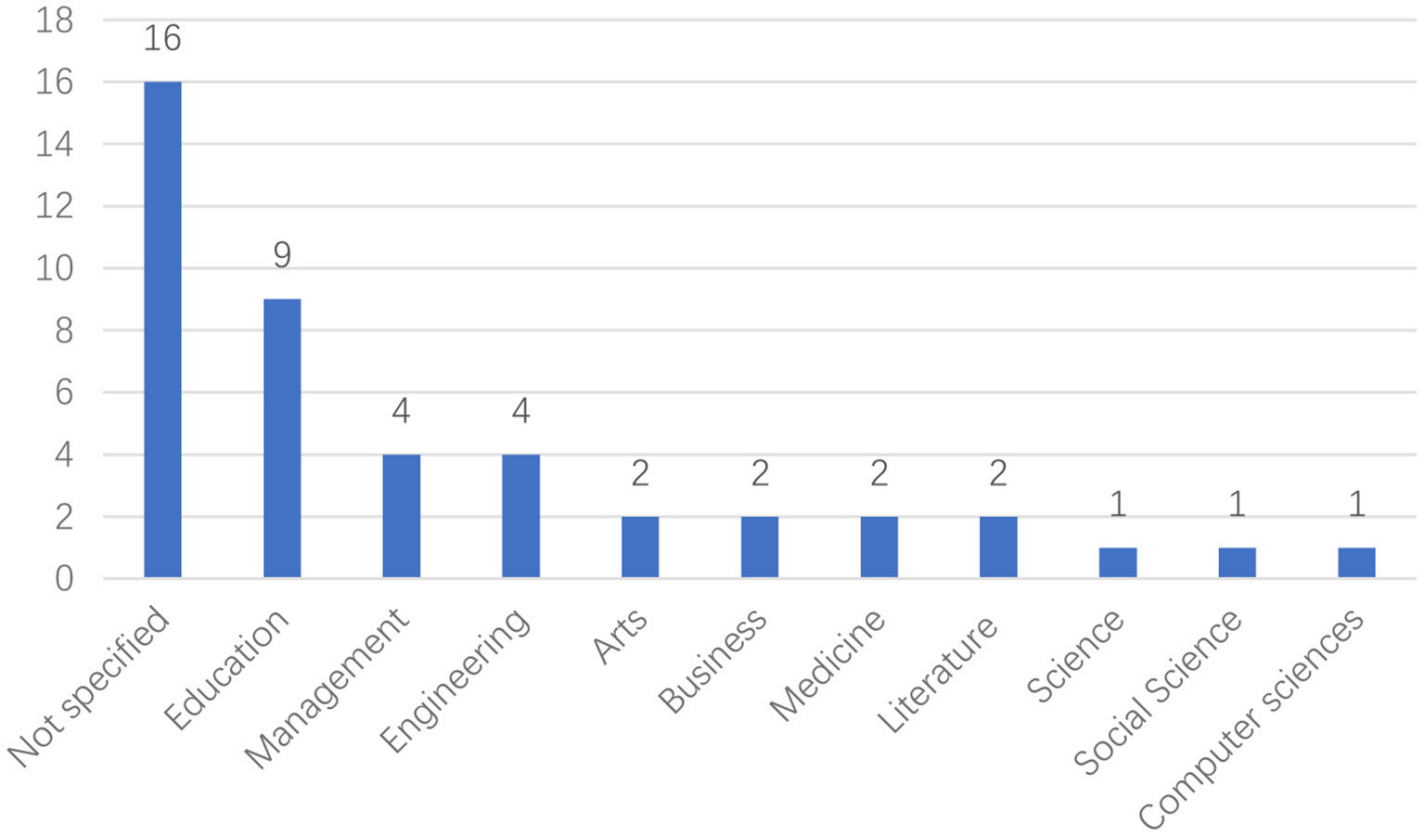
Distribution of participants' major.

The present study starts this section with the results related to publication year, languages, and participants. Figure 3 shows the distribution of articles published in the past 10 years. Most studies were published in 2019 (*n* = 8), and no articles were published in 2012. From the figure, it can also be seen that the research trend in this field is on the rise. Figure 4 demonstrates the frequency at which different languages were the focus in the reviewed studies. 29 studies focused on English. There were also studies focused on Chinese (*n* = 2), Ukrainian (*n* = 1), Japanese (*n* = 1), and Spanish (*n* = 1). As shown in Figure 5, undergraduates were the most common academic level (*n* = 17), and there was a relatively low number of studies conducted on junior high school (*n* = 5), senior high school (*n* = 2), and primary school (*n* = 1) academic levels. As shown in Figure 6, researchers were more willing to involve students who were majoring in the fields of education (*n* = 9), management (*n* = 4), or engineering (*n* = 4).

### Research Focus

**Figure 7:**
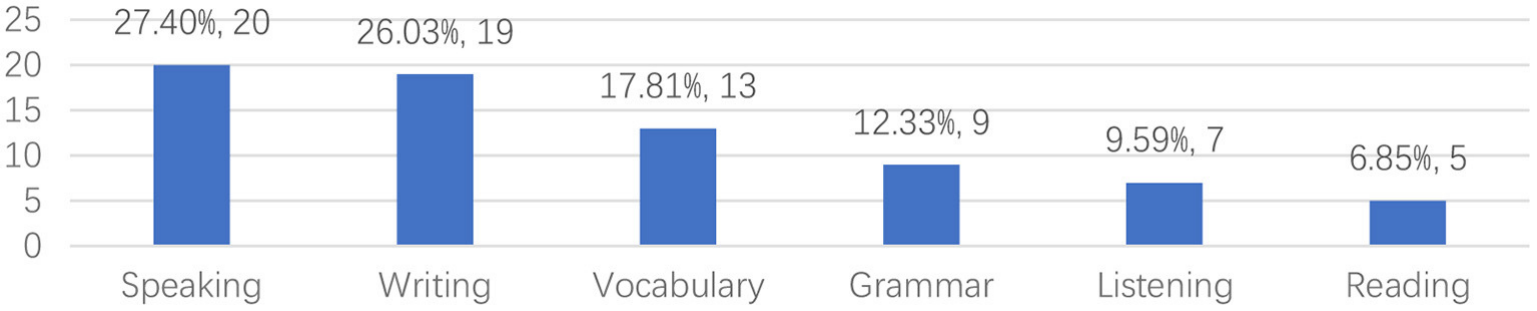
Distribution of language skills.

This section presents the results related to research focus of reviewed articles. As can be seen from Figure 7 (and from Appendix 1), researchers carried out technology-assisted language learning studies and focused on the development of listening, speaking, reading, writing, grammar, and vocabulary skills. Among these skills, speaking skills (*n* = 20) received considerable attention from researchers, followed by writing skills (*n* = 19) and vocabulary (*n* = 13). Reading (*n* = 5) skills received less interest from researchers.

**Figure 8:**
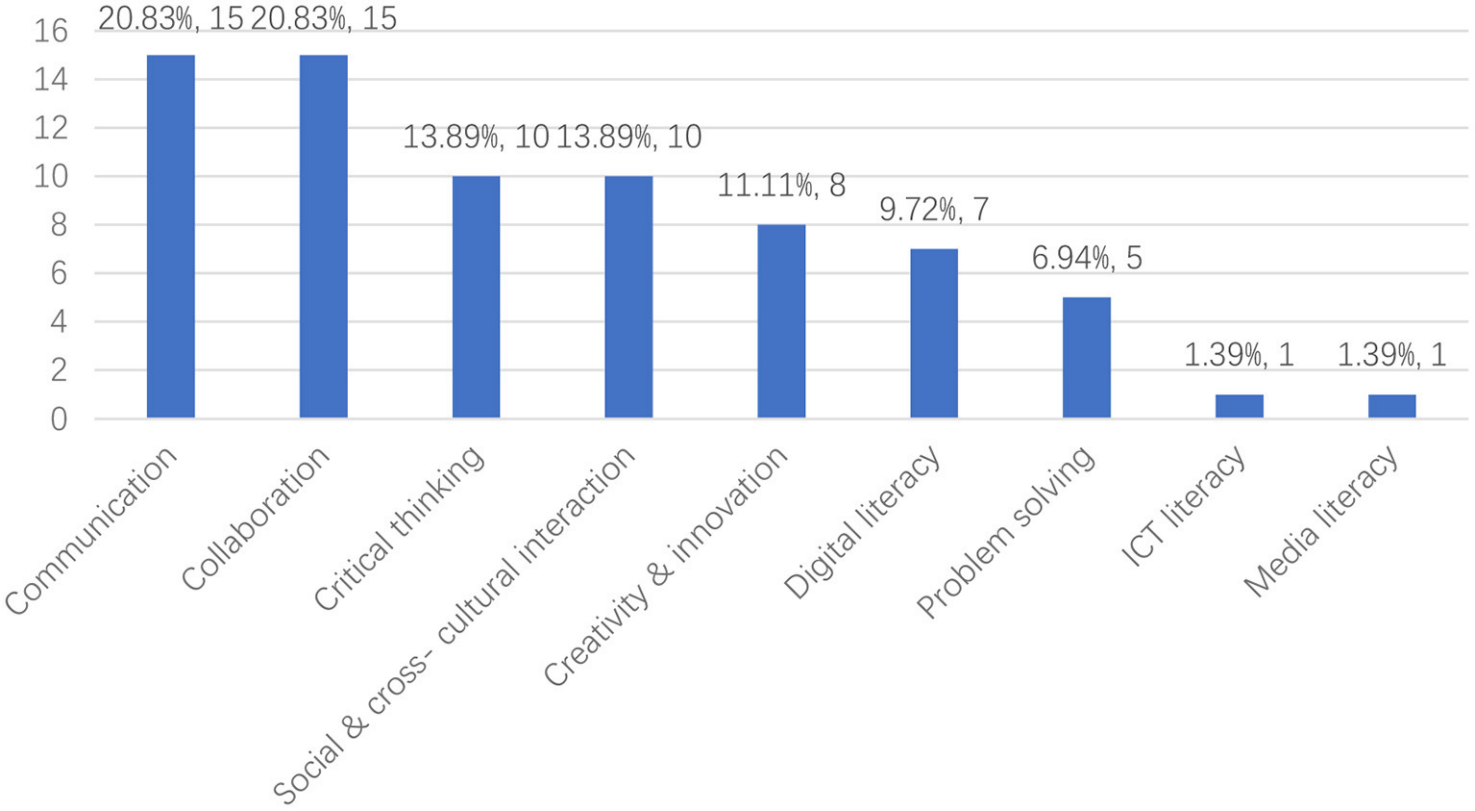
Distribution of 21st century skills.

According to Figure 8 (and Appendix 1), researchers pointed out that technology-supported language learning can also promote 21st century skills. These skills relate to the following three categories: 4C (communication, collaboration, critical thinking, and creativity), digital literacy, and career and life skills. The most common skills that scholars targeted were communication (*n* = 15) and collaboration (*n* = 15), followed by critical thinking (*n* = 10) and social and cross-cultural interaction (*n* = 10). Problem solving (*n* = 5) skills have received the least attention from researchers.

### Theoretical Foundation

This section focuses on theoretical foundation in the reviewed articles. As shown in Appendix 2, a total of 16 theories were used. The most used theory was the social constructivism theory (*n* = 9), followed by Byram's intercultural competence model (*n* = 3), project-based learning (*n* = 2), content based instruction (*n* = 2), task based approach to language teaching (*n* = 2), and sociocultural theory (*n* = 2). The rest of theories were used only once.

### Technology

As shown in Appendix 3, a total of 52 technologies were used in reviewed studies. This review grouped them into eight categories: Social tools (*n* = 20), Creative tools (*n* = 19), Collaboration tools (*n* = 13), Learning management system (*n* = 9), Multimedia materials (*n* = 5), Classroom interactive tools (*n* = 4), Presentation tools (*n* = 2), Wearable devices (*n* = 1). Among the most commonly used technologies were Facebook (*n* = 4), Google Docs (*n* = 4), Moodle (*n* = 4), followed sequentially by Skype (*n* = 3), Padlet (*n* = 3), WhatsApp (*n* = 2), YouTube (*n* = 2), Blogs (*n* = 2), Google Drive (*n* = 2), and Wiki (*n* = 2). The other 40 technologies have only been used once, i.e., Windows Movie Maker, Live On, Edmodo, Kahoot, and Prezi. In addition, one study involved a virtual reality production tool (EduVenture) and a wearable device (Google Cardboard).

### Learning Activity

As shown in Appendix 4, in reviewed studies, scholars designed the following five main types of learning activities: (1) collaborative task-based language learning (*n* = 9); (2) learning activities based on online communication (*n* = 9); (3) creative work-based language learning (*n* = 8); (4) adaptive learning activities (*n* = 4); and (5) learning activities based on multimedia materials (*n* = 4).

### Methodology

This section presents methodological details of reviewed studies, such as sample size, research duration, data collection tools and research design.

**Figure 9:**
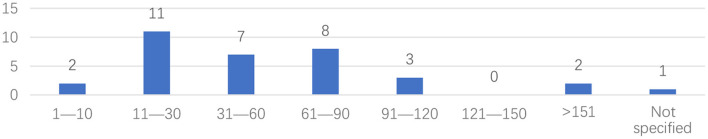
Sample size distribution.

**Figure 10:**
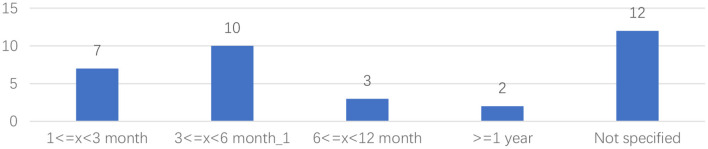
Research duration distribution.

As shown in Figure 9, the most common sample size was from 11 to 30 participants (*n* = 11), followed by sample sizes between 61 and 90 (*n* = 8) and between 31 and 7 (*n* = 7). Only two studies selected a sample size between 1 and 10. The sample size of two studies was >151. As shown in Figure 10, most of research duration was between 3 and 6 months (*n* = 10). There were 12 studies that did not state any research duration.

As shown in Appendix 5, the most common data collection method was questionnaires (*n* = 17), followed by tests (*n* = 15) and interviews (*n* = 13). Two data collection methods were used only 2 times, they were scales (*n* = 2) and rubric (*n* = 2).

As shown in Appendix 6, research designs related to technology-supported language learning and 21st century skills were categorized into three main categories, namely quasi-experimental research (*n* = 14), case studies (*n* = 12), and action research (*n* = 8).

### Findings

As shown in Appendix 7, various findings were reported in reviewed studies. In addition, that learners' language skills acquisition and 21st century skills, technology-supported language learning activities provided learners with good learning experiences, enhanced motivation and engagement, and improved self-confidence. In reviewed studies, some scholars reported about challenges faced by students during learning activities; they included challenges from technology, from their own competence, challenges of collaborating with others and self-attitude.

## Discussion

### Research Focus

#### Language Skills

Regarding language skills, researchers have focused on improving learners' speaking, writing and vocabulary skills more. This shows that researchers are more concerned with the improvement of learners' skills related to language output. Researchers who reviewed studies on technology-supported language learning from 2014 to 2019 came to the same conclusion (Shadiev and Yang, 2020). However, the present study showed that reading skills received the least attention, while previous studies noted that grammar skills received less attention. This revealed that researchers are now beginning to pay more attention to previously neglected skills and are beginning to focus on the role of technology-supported language learning in facilitating learners' grammar skills. For example, Lai (2017) noted that grammar skills improved when learners completed activities to create vocabulary lists and greeting cards using multimedia resources. Jung et al. (2019) noted that students' grammar skills improved as they corrected each other's pronunciation and grammatical errors through video chat. Jamalai and Krish (2021) found that students' grammar skills improved through online forum discussions and knowledge sharing.

#### 21st Century Skills

In terms of 21st century skills, communication and collaboration have received the most attention from researchers. It is probably because the 21st century society is more globalized and along with the increased complexity of related work, interpersonal communication and cooperation are being enhanced. The 21st century society emphasizes teamwork skills, and therefore scholars focus on collaborative and communication skills. Problem-solving skills have received little attention, and no researcher focused on career and life skills. In the face of the evolving and changing society of the future, problem-solving skills are among the core 21st century skills, emphasizing learners' ability to define problems, think critically, and solve problems. For example, scholars in reviewed studies have focused on learners' problem-solving skills in virtual technology-supported language learning (Chen et al., 2021).

Based on the results, this study has several recommendations for educators and researchers. First, input skills are an important component of language skills and an indispensable way for learners to develop output skills (Harmer, 2007). The present study suggests that researchers can focus on learners' input skills supported by technology, such as listening and reading. Second, problem-solving skills and career and life skills also deserve attention; therefore, future studies try to explore the effects of technology-supported language learning on these skills.

### Theoretical Foundation

#### Theories Related to Instructional Design

The most commonly used instructional design theory in reviewed studies was social constructivism theory. The results of this research are consistent with those of previous review studies of technology-supported language learning (Parmaxi and Zaphiris, 2017). According to this theory (Vygotsky, 1978), knowledge is not a set of “facts” but rather a synthesis of information that is actively constructed and evolving in the learner's mind. The teacher does not “give” knowledge to the learner, but the learner should acquire knowledge actively. Learners' knowledge evolves as they process old and new information, as well as their experiences. The researchers designed collaborative, creative, and communicative activities based on a social constructivism perspective to encourage learners to input the target language and output the target language in a meaningful context. At the same time, researchers have used various learning and teaching activities to promote students' collaboration, communication, creativity, critical thinking and digital literacy skills (Yang et al., 2013, 2014, 2022; Lai, 2017; Sevilla-Pavón and Nicolaou, 2017; Huang, 2021).

Other researchers have also used theories based on learner-centered pedagogies such as problem-based or project-based theories. These pedagogies are all used to promote student-directed learning, adaptive learning, and personalized assessment. Learning theories were used to design activities that provided learners with opportunities for language input and output, e.g., to learn new knowledge and then apply it to the real world by creating own content. This allows learners to acquire language skills and develop 21st century skills such as communication, collaboration, and problem solving (Arnó-Macià and Rueda-Ramos, 2011; Yang et al., 2013, 2014; Srebnaja and Stavicka, 2018).

#### Theories Related to Language Learning

Researchers have also designed learning activities based on theories related to foreign language learning, such as task-based language teaching, content-based instruction, and output-input theory. For example, digital story creation activities and integrated cross-cultural communication activities designed by the researcher are in line with these theoretical perspectives, in which learners have access to the target language through social tools and partner communication. The ability to use creative and collaborative tools to complete target-language based tasks also contributes to the acquisition of language skills and 21st century skills development, such as social and cross-cultural interaction, communication skills (Lewis and Schneider, 2015; Tseng, 2017).

#### Theories Related to Measuring Learning Outcomes

Since language learning is closely related to culture, scholars have designed foreign language courses based on cross-cultural communication, where learners acquired both language skills and cultural knowledge. Further, there are theories that have been used by scholars to assess and measure learners' outcomes. For example, researchers have focused on learners' intercultural competence along with their language skills and utilized the Byram' ICC model and the developmental model of intercultural sensitivity to measure their cross-cultural knowledge acquisition and skills development (Bennett, 1986; Byram, 1997). In addition, the Keller' ARCS motivational model (Keller, 1987) has been used by researchers to measure learners' perceived attention, relevance, confidence, and satisfaction in technology-supported language learning environments.

This review analyzed the theoretical foundation that was used by those few studies that focused on non-English languages such as Chinese, Ukrainian, Japanese, and Spanish. This review found that learning theories used by scholars in these studies were diverse. They were related to instructional design (e.g., social constructivism), language learning (e.g., language output and input), and cross-cultural learning (e.g., intercultural sensitivity).

Based on the findings, several suggestions for educators and researchers are proposed. First, the theories mentioned by researchers are instruction-related theories, language learning-related theories, and measurement-related theories; they were used to guide the design of technology-supported language learning activities that focus both on the acquisition of language skills and on the 21st century skills. These theories can be useful to inform the design of appropriate language learning activities for educators and researchers in the future. Second, this review found that many researchers did not indicate what theories were used in their studies. Theoretical foundations are important for the instructional design, language learning or measuring activities, so such information should be clearly indicated so that other researchers can gain a deeper understanding of them.

### Technology

#### Eight Technologies With Different Functions

Based on the literature review, this study grouped technologies into eight categories based on their functions: (1) collaborative tools (e.g., Google Docs or Padlet) for supporting learners to collaborate on a task through co-editing and information sharing; (2) social tools (e.g., Facebook or Skype) for supporting learners to communicate and share content remotely or synchronously using text, audio and video; (3) creative tools (e.g., Photo Story or Adobe Spark) to support learners in creating work, such as digital stories or videos; (4) learning management system (e.g., Moodle) to integrate learning activities and learning resources for adaptive online learning; (5) classroom interaction tools (e.g., Quizlet or Kahoot) to support question-answering, polling, and other activities in the classroom; (6) multimedia materials are some audio and video resources on the web or multimedia textbooks; (7) presentation tools (e.g., PowerPoint) are used to support learners to present their learning content digitally; (8) wearable devices (e.g., Google Glass) to support learners to view or interact with content in virtual reality learning environments.

#### Most Commonly Used Technologies

Facebook (social tool), Google Docs (collaboration tool), and Moodle technologies (learning management system) were used the most in previous studies to facilitate language and 21st century skills. The study further analyzed which technologies are most often used by researchers to promote 21st century skills. Appendix 8 demonstrates these most commonly used tools. The study found that Facebook (social tool), Google Docs (collaboration tool) and Moodle (learning management system) were also the tools most often used by researchers to promote communication, collaboration and critical thinking, social and cross-cultural interaction skills. This indicates that scholars valued such 21st century skills as collaboration and communication among students in technology-supported language learning activities. For example, Sevy-Biloon and Chroman (2019) used social and collaborative tools (e.g., Google Docs, Facebook, etc.) to support communication between students from different cultural backgrounds and their results showed that students' speaking skills, social and cross-cultural interaction, and communication skills were promoted. Moodle is popular among researchers because this learning management system not only supports learners' adaptive and inquiry-based learning, but also helps teachers share learning resources with learners, design learning activities, and manage learners' learning progress (García-Sánchez and Burbules, 2016). For example, Yang et al. (2014) designed a language learning activity based on the Moodle platform that asked students to complete reading and writing tasks in the system to promote the development of reading, writing skills and critical thinking. In addition, researchers most often used Google Docs (collaboration tool), Prezi (presentation tool), Windows Movie Maker, Photo Story3 (creative tools) and Blogs (social tool) to support students' creativity and innovation skills, problem-solving skills, and ICT literacy. And only two studies have used films (multimedia materials) and blogs (social tool) to support students' media literacy.

#### Experienced Challenges of Using Technology

Scholars reported that technologies pose some challenges for learners. For example, students were not experienced to use technology and had no trainings before learning activities; then they complained about problems to use technology during learning (Lai, 2017). Students were also confused about the layout of the platform and noted that there were incompatibilities and connectivity issues with learning devices (Hosseinpour et al., 2019). When communicating remotely, students pointed out that there were problems with the network and they were not able to connect and participate in learning process (Mohamadi Zenouzagh, 2018; Jung et al., 2019).

#### The Distribution of Technology in non-English Language Studies and Different Theories

This review also analyzed technologies that were used by those few studies that focused on non-English languages. This review found that, in general, scholars in these studies used such technologies as creative tools (Adobe Spark), collaboration tools (Google Docs), and social tool (Facebook) to present multimedia content to learners and support collaborative, creative and communicative learning activities (Valdebenito and Chen, 2019).

With regard to the distribution of technology in theory. Social constructivism theory was the most commonly cited theory in reviewed research and scholars used various technologies such as learning management systems (e.g., Moodle), creative tools (e.g., iMovie) or social tools (e.g., Facebook) to support constructivism-based learning activities. That is, interactive and collaborative learning activities were designed for students to learn new knowledge and then apply it to construct meaning in authentic contexts.

Based on the results of this study, several recommendations for educators and researchers were proposed. First, it is recommended that learning activities supported by technologies are designed based on appropriate theoretical foundation. Second, teachers are encouraged to conduct appropriate technology training for students beforehand so that they become familiar with technology tools. Third, teachers and researchers should test learning tools with students in advance in order to identify any possible technical problems, and provide timely support during learning process.

### Learning Activities Used to Promote Language Skills and 21st Century Skills

This section describes what technologies are used in each type of learning activity and how they contribute to the development of learners' language skills and 21st century skills. In addition, it offers relevant suggestions to researchers and educators.

#### Adaptive Language Learning Activities on Learning Platforms

As shown in Table 1, in the reviewed study, researchers used the following tools: Moodle, Google classroom, Quantum leap, and WebQuest, to develop adaptive language learning activities on learning platforms. These tools are used to integrate different types of instructional resources and diverse language learning activities to provide learners with adaptive learning materials that meet their learning needs. Students can ask questions and receive feedback from other students or teachers, and take control of their own learning progress.

**Table 1:** Adaptive language learning activities on learning platforms.

**References**	**Technology**	**Learning activity**	**Language skill**	**21st century skills**
Arnó-Macià and Rueda-Ramos (2011)	Learning management system: Quantum LEAP	Online learning, complete listening, speaking, and reading tasks	Listening Speaking	Critical thinking
Yang et al. (2013)	Learning management system: Moodle	Online learning, complete speaking and listening tasks	Listening Speaking	Critical thinking
Yang et al. (2014)	Learning management system: Moodle	Online learning, complete reading, and writing tasks	Reading Writing	Critical thinking
Srebnaja and Stavicka (2018)	Learning management system: WebQuests	Online learning, complete speaking and writing tasks	Speaking Grammar Writing	Creativity and innovation, collaboration, communication, digital literacy

For example, Arnó-Macià and Rueda-Ramos (2011) designed tasks for reading, listening, and speaking practice in Quantum leap platform. Researchers have designed listening tasks in Moodle platform; students were required to analyze, evaluate, and summarize content after listening (Yang et al., 2013, 2014). Srebnaja and Stavicka (2018) designed WebQuests-based speaking and writing tasks.

All of these studies noted that learners' performance in speaking, listening, reading, writing, and grammar improved after completing the computer-assisted adaptive language learning tasks. In addition, students' critical thinking skills were developed.

#### Collaborative Task-Based Language Learning Activities

As shown in Table 2, the following tools were used by researchers for the development of collaborative-based language learning activities: (1) collaboration tools: Google Docs, Google Drive, Wiki, Edmodo, and E-writing forum. These collaborative tools have the following functions: sharing, collaborative editing, cloud storage, synchronized display, and help students freely share information in various formats (e.g., text, images, videos, web links, audio recordings, music, etc.) on the platform so that they can exchange ideas and collaborate on editing content; (2) creative tools: Adobe Spark, to support students' expression of ideas; (3) social tools: Blogs or WordPress, to support students in reading and commenting on each other's work.

**Table 2:** Collaboration-based language learning activities.

**References**	**Technology**	**Learning activity**	**Language skill**	**21stcentury skills**
Amir et al. (2011)	Social tools: Blog	Collaborate on writing tasks	Writing	Collaboration
García-Sánchez and Burbules (2016)	Learning management system: Moodle Collaboration tools: Wiki	Students propose solutions to social problems	Speaking Vocabulary	Communication Collaboration Digital literacy Problem solving
Lai (2017)	Collaboration tools: Padlet Creative tools: Home Styler, Thing Link	Collaborate on different tasks, such as creating vocabulary list, greeting cards	Vocabulary Grammar	Collaborative Communication
Mohamadi Zenouzagh (2018)	Collaboration tools: E-writing forum	Collaborate on writing tasks	Writing	Collaboration
Valdebenito and Chen (2019)	Creative tools: Adobe Spark, Google My Maps Collaboration tools: Google Doc, Word Press	Collaborate on culture tasks	Listening Speaking Writing vocabulary Grammar	Critical thinking Digital literacy Collaboration Communication
Huh and Lee (2020)	Collaboration tools: Google Docs	Cooperate to complete role plays or songs to express the vocabulary learned	Speaking Writing	Creativity and innovation
Hosseinpour et al. (2019)	Collaboration tools: Edmodo	Collaborate on writing tasks	Writing	Collaboration
Girgin and Cabaroglu (2021)	Classroom interactive tools: Quizlet, Quizizz, Cram, Kahoot Creative tools: Story Bird, Voki, Go Animate, Animoto, Powtoon, Canva, Poster MyWall Collaboration tools: Padlet	Watch the video Collaborating on classroom tasks Creating digital stories Share and communicate	Listening Speaking Reading Writing Grammar vocabulary	Collaboration Critical thinking Creativity and innovation communication
Chen et al. (2021)	Creative tools: Edu Venture Wearable devices: Google Cardboard	Solve problems and create videos collaboratively	Vocabulary	Problem solving

Collaboration-based language learning activities are those in which students work in groups to solve problems and complete tasks proposed by the teacher, such as asking students to provide an essay or present their ideas in other ways (e.g., a solution, a report, and a performance). For example, Amir et al. (2011) asked students to work in groups to publish six articles based on different topics over the course of 14 weeks, and one of the tasks required students to find and discuss software about computer-assisted writing.

Mohamadi Zenouzagh (2018) designed a collaborative writing activity based on the E-writing platform. Valdebenito and Chen (2019) designed a collaborative activity on the theme of “food and culture” in which students first had to use Google Maps to identify geographic areas related to the content, then use a Google Doc to record their ideas, and finally use video production tools such as Adobe Spark to express their ideas and share them on the WordPress platform. Huh and Lee (2020) designed a creative learning English collaborative activity in which students first used a mobile app to learn how to spell words, then the group took the words they learned and expressed them through the role play and song. Lai (2017) designed different collaborative tasks, for example, students needed to use the ThingLink tool to create vocabulary lists and greeting cards related to the topic, which were then shared on the Padlet platform and discussed. In addition, students were required to use HomeStyler to collaboratively design a dream home and use some vocabulary related to “location” to describe the design of their home.

Girgin and Cabaroglu (2021) designed an English learning project that integrates Web 2.0 technology and flipped classroom, and students used Padlet to watch videos in class. In grammar classes, students used Kahoot, Quizlet, Quizizz, Animoto, Powtoon, and Poster MyWall to answer grammar questions. In vocabulary and reading classes, students used tools such as Mind Meister, Voki, Canva, Cram, Go Animate and Story-bird to create mind maps, as well as create digital stories, which can be presented and shared. Chen et al. (2021) used virtual reality technology to design language learning activities. Learners were required to first watch a virtual reality scene and think about how to solve the problem based on a series of guiding questions provided by the teacher. Then students role-played in English to create a virtual reality video of the problem being solved.

The results of the abovementioned studies showed that collaborative-based language learning activities facilitated the development of learners' language skills. The researchers noted that collaborative problem-solving language learning activities provided learners with a large number of writing tasks, such as writing reports, essays, or creating storylines and designing works. The process of sharing with each other enabled to point out grammatical errors (Amir et al., 2011; Mohamadi Zenouzagh, 2018; Hosseinpour et al., 2019). When learners used multimedia resources to create vocabulary lists and greeting cards, their vocabulary and grammar skills were also improved (Lai, 2017).

At the same time, students' critical thinking was developed as they gave each other's critical and constructive comments (Valdebenito and Chen, 2019; Zou and Xie, 2019; Girgin and Cabaroglu, 2021). In addition, students completed tasks in small groups which promoted the development of communication and collaboration skills during discussions with each other (Amir et al., 2011; García-Sánchez and Burbules, 2016; Lai, 2017; Mohamadi Zenouzagh, 2018; Hosseinpour et al., 2019; Zou and Xie, 2019; Girgin and Cabaroglu, 2021). The process of students voicing digital content promoted the development of speaking skills (Huh and Lee, 2020). In the process of creating digital works, digital literacy was developed (García-Sánchez and Burbules, 2016; Valdebenito and Chen, 2019). Chen et al. (2021) pointed out that learners learn contextually in an immersive learning environment, and solving real problems through virtual reality technology improved learners' vocabulary as well as promoted their problem-solving skills.

#### Creative Work-Based Language Learning Activities

As shown in Table 3, in reviewed studies, language learning activities based on creative works consisted of two main categories: creating digital stories or videos. The main models for this type of learning activity were as follows: students communicated in groups about how to create a digital story or video, then collected and processed relevant information, after that created a digital story, and finally shared content and communicated with each other about it.

**Table 3:** Creative work-based language learning activities.

**References**	**Technology**	**Learning activity**	**Language skill**	**21st century skills**
Thang et al. (2014)	Creative tools: Photo Story3 Social tools: Blog	Create digital stories and share	Writing Speaking	Communication Creativity and innovation Collaboration ICT literacy
Sevilla-Pavón and Nicolaou (2017)	Creative tools: iMovie, Inspiration Collaboration tools: Google Docs, Google Drive, Facebook, WhatsApp Presentation tools: PowerPoint, Prezi Social tools: Google+ Community, Google+ Forum	Create digital stories and share	Speaking Listening Reading Writing Vocabulary	Communication Collaboration Creativity and innovation Critical thinking Problem solving Digital literacy Social and cross-cultural interaction
Kulsiri (2018)	Creative tools: Windows Movie Maker	Creative video	Speaking Reading Writing Vocabulary	Creativity and innovation Collaboration Problem-solving
Yalçin and Öztürk (2019)	Learning management system: Google-classroom	Rewrite story endings, create digital stories and share	Writing	Communication Collaboration Creativity and innovation
Chiang (2020)	Creative tools: Story Bird	Create digital stories and share	Writing	Digital literacy
Yang et al. (2022)	Creative tools: Audacity Collaboration tools: Google Drive Presentation tools: Prezi	Create digital stories and share	Speaking	Creativity and innovation
Mirza (2020)	Social tools: YouTube Presentation tools: PowerPoint	Create digital stories and share	Speaking	Communication
Huang (2021)	Creative tools: Smartphone camera	Smartphone-based video creation	Speaking	Communication Digital literacy

The researchers chose different tools to support such learning process, e.g., (1) creating digital stories, i.e., Photo Story3, Windows Movie Maker, or iMovie; (2) creating video scripts in collaboration, i.e., Google Docs or Google Drive; (3) presenting digital stories, i.e., Prezi or PPT; (4) sharing digital stories and communicating, i.e., Google+ forums, Facebook, Instagram, WhatsApp, Google Classroom, and classroom management systems.

The researcher noted that digital storytelling promoted language skills, specifically, the process of writing story scripts promoted students' writing and vocabulary skills (Thang et al., 2014; Sevilla-Pavón and Nicolaou, 2017; Kulsiri, 2018; Yalçin and Öztürk, 2019; Chiang, 2020). It also promoted 21st century skills. Researchers mentioned three approaches for creating digital stories or videos such as free-writing, rewriting the ending of the story, and specifying the theme, and in this open-ended work creation process, students' sense of creativity, problem-solving skills, and digital literacy were developed (Thang et al., 2014; Sevilla-Pavón and Nicolaou, 2017; Kulsiri, 2018; Yalçin and Öztürk, 2019; Yang et al., 2022). Regarding the creation of digital stories on a specific theme, the researcher asked learners to design a new country, and students needed to understand a range of elements including different countries and cultures, such as national characteristics, language, national policies, climate and life. As a result, students' social and cross-cultural skills were improved. In addition, critical thinking was facilitated as students developed different ideas and perspectives as they evaluated each other's digital stories (Sevilla-Pavón and Nicolaou, 2017). Finally, students developed their communication and collaboration skills when working in groups (Thang et al., 2014; Sevilla-Pavón and Nicolaou, 2017; Kulsiri, 2018; Yalçin and Öztürk, 2019; Mirza, 2020; Huang, 2021).

#### Language Learning Activities Based on Multimedia Learning Materials

As shown in Table 4, language learning activities based on multimedia materials involved such tools as (1) web-based learning management system, e.g., EDpuzzle; (2) social tool, e.g., YouTube; and (3) multimedia textbooks. All of them provided multimedia resources for students. There were also (4) collaboration tools, e.g., Padlet and Google docs, which supported learners to share ideas with each other.

**Table 4:** Language learning activities based on learning multimedia materials.

**References**	**Technology**	**Learning activity**	**Language skill**	**21st century skills**
Tseng (2017)	Multimedia materials	Watch multimedia materials oral report and reflection on cultural differences	Listening	Social and cross-cultural interaction
Zou and Xie (2019)	Learning management system: EDpuzzle Collaboration tools: Google Docs, Padlet	Watch video on writing skills, discuss in small groups and complete a report	Writing	Critical thinking Collaboration
Nikitova et al. (2020)	Multimedia materials: Multimedia textbooks	Study multimedia materials and complete tasks	Speaking Writing Grammar Vocabulary	Collaboration Critical thinking Communication Problem solving
Aristizábal-Jiménez (2020)	Multimedia materials: video	Watch YouTube videos and analyze, make videos and presentation	Vocabulary Grammar	Critical thinking

Scholars have designed a variety of language learning activities based on multimedia materials, but the topics and learning tasks of the multimedia materials involved in these studies differed. For example, Tseng (2017) asked learners to watch a video on the topic of cultural differences, and then students gave oral presentations and reflections to present their views on cultural differences. Zou and Xie (2019) asked students to watch a video on EDpuzzle, then to discuss in groups, negotiate and compare answers, to share their output to the Padlet platform, and finally submit their reports in Google docs. Nikitova et al. (2020) asked students to watch videos from multimedia textbooks with different English contexts and then simulated learners' role play activities. Aristizábal-Jiménez (2020) asked learners to watch YouTube videos, analyze the structure and content of video content, and then create posters to present and share their ideas.

The researcher noted that language learning activities based on multimedia materials promoted learners' language skills and 21st century skills. Specifically, learners' listening skills were promoted after watching the videos (Tseng, 2017). Culturally relevant content in videos and culture-based communication among peers promoted students' social and cross-cultural interaction skills (Tseng, 2017). Learners actively used dictionaries and discussed grammar while completing tasks to make the information easier to understand, which also promoted students' vocabulary and grammar skills (Aristizábal-Jiménez, 2020). In addition, working in groups to complete tasks promoted speaking, writing, grammar, and vocabulary skills. This was also beneficial to develop students' problem solving, collaboration, critical thinking, and communication skills (Aristizábal-Jiménez, 2020; Nikitova et al., 2020).

#### Language Learning Activities Based on Online Communication

As shown in Table 5, the researchers designed online communication-based language learning activities. Most of them were cross-cultural communication activities to support cross-cultural communication between students from different cultural backgrounds. In terms of technology, the researchers mainly used social tools to support textual or video communication, e.g., Facebook, Skype, and WhatsApp. In addition, researchers have utilized learning management systems to support students to view learning resources uploaded by teachers.

**Table 5:** Language learning activities based on online communication.

**References**	**Technology**	**Learning activity**	**Language skill**	**21st century skills**
Calogerakou and Vlachos (2011)	Multimedia materials: Film Social tools: Blog	Students from different cultural backgrounds watch films with culturally relevant backgrounds and communicate	Writing	Social and cross-cultural interaction Media literacy
Chen and Yang (2014)	Social tools: ePals, iEARN, Skype	Students from different cultural backgrounds share culturally specific folklore stories, make videos, and perform puppet shows	Writing Vocabulary	Social and cross-cultural interaction Communication Collaboration
Lewis and Schneider (2015)	Social tools: Skype	Students from different cultural backgrounds discuss cultural topics online	Speaking Grammar	Social and cross-cultural interaction Communication
Chen and Yang (2016)	Learning management system: Moodle Social tools: Wiki	Students from different cultural backgrounds discuss movies with culturally diverse content online	Speaking Reading Writing Vocabulary	Social and cross-cultural interaction
Özdemir (2017)	Social tools: Facebook, YouTube	Watch YouTube videos and discuss online based on cross-cultural questions prepared by the instructor	Writing Listening	Social and cross-cultural interaction
Sevy-Biloon and Chroman (2019)	Social tools: Facebook, Skype, WhatsApp, Facetime	Students from different cultural backgrounds discuss cultural topics online	Speaking	Communication Social and cross-cultural interaction
Jung et al. (2019)	Social tools: Live On	Students from different cultural backgrounds discuss cultural topics online	Grammar Vocabulary Speaking	Social and cross-cultural interaction
Hirotani and Fujii (2019)	Social tools: Facebook	Students from different cultural backgrounds exchange proverbs online, write reflection journals and perform skits reflecting on cultural differences	Speaking Grammar	Communication Social and cross-cultural interaction
Jamalai and Krish (2021)	Social tools: online forum (not specific)	Online topic discussion	Grammar Vocabulary Speaking	Critical thinking Digital literacy

The design of cross-cultural communication activities followed the same pattern—exposure to cross-cultural knowledge, reflection on cross-cultural differences, and cross-cultural exchange. For example, Calogerakou and Vlachos (2011) had students from two countries to watch movies and compare culture presented in movies with their own culture. Then students had to post comments on a blog and discuss their ideas. Chen and Yang (2016) asked students to share culturally specific folklore stories with their partners and to make videos of the stories to send to their partners. In addition, students were asked to perform a puppet show *via* videoconference. All of these were for students to learn about cultural similarities and differences. Chen and Yang (2014) designed a discussion activity based on cultural themes; for example, students discussed movies that involved culturally different content, and then students shared their opinions on Wiki. Lewis and Schneider (2015) asked learners to interact with native Spanish-speaking students and discuss cultural topics such as “local living conditions” and “how to celebrate holidays.” Learners were then asked to write a mini-biography or travel brochure for their study partner to demonstrate the cultural knowledge they gained during the exchange. Özdemir (2017) asked students to watch YouTube videos and discuss them based on cross-cultural questions prepared by the teacher. Sevy-Biloon and Chroman (2019) designed an intercultural exchange program in which students from Ecuador and the United States were randomly paired and then engaged in a cultural exchange based on the theme of the language course. Jung et al. (2019) asked students from different cultural backgrounds to discuss cultural topics, including “happiness factors, family, and food,” and finally, students reflected on the discussion, exchanged proverbs with each other, and then presented cultural differences. They reflected on their experiences in a reflective journal. Jamalai and Krish (2021) designed an online discussion activity, in which learners were required to engage in online discussions based on topics posted by teachers in a forum.

The results showed that students' speaking, vocabulary, writing, reading, and grammar skills improved when communicating through text and speech because students double-checked vocabulary spelling and grammar. Students identified errors they made when communicating using text and speech and corrected them to ensure that others understood their intended meaning (Calogerakou and Vlachos, 2011; Chen and Yang, 2014, 2016; Lewis and Schneider, 2015; Özdemir, 2017; Hirotani and Fujii, 2019; Jung et al., 2019; Sevy-Biloon and Chroman, 2019; Jamalai and Krish, 2021). In addition, students' listening skills improved after watching YouTube videos (Özdemir, 2017).

At the same time, students' communication process using social tools developed the ability to use writing software, electronic dictionaries, and collect information on the Internet, and therefore media literacy was improved (Calogerakou and Vlachos, 2011). All studies point to the development of cultural interaction skills after students interacted and exchanged different cultural perspectives with partners (Calogerakou and Vlachos, 2011; Chen and Yang, 2014, 2016; Lewis and Schneider, 2015; Özdemir, 2017; Hirotani and Fujii, 2019; Jung et al., 2019; Sevy-Biloon and Chroman, 2019). Communication (Chen and Yang, 2014; Lewis and Schneider, 2015; Hirotani and Fujii, 2019) and collaboration skills were also developed (Chen and Yang, 2014) in reviewed studies.

This review also analyzed learning activities that were used by those few studies that focused on non-English languages. This review found that most learning activities designed in these studies were online cross-cultural communicative activities. This shows that the primary goal of these learning projects was to develop students' foreign language and intercultural communication skills.

Based on the findings of the reviewed literature, the five types of language learning activities supported by technology had a positive impact on students' language skills as well as their 21st century skills development. Moreover, this review found that these learning activities followed similar pattern. The common pattern for language learning activities based on culture-related communication was exposure to cross-cultural knowledge, reflection on cross-cultural differences, and cross-cultural exchange. The common pattern of language learning activities for creative works was as follows: students communicated in groups about how to create a work (such as digital story or video), then collected and processed relevant information, created a work, and then shared content and communicated with each other about it. These patterns could provide suggestions for researchers and teachers to design similar instructional activities that target development of language skills and 21st century skills in the future.

Second, this review found that researchers designed similar instructional activities, but the research focus was different. For example, in the adaptive language learning activities on learning platforms, researchers focused on the development of students' speaking skills and lacked attention to reading skills. And in the collaborative task-based language learning activities, researchers have focused more on writing and vocabulary skills, collaboration, and communication skills, and lacked attention to listening skills. In creative writing-based language learning activities, researchers focused more on speaking and writing skills as well as creative and communication skills.

### Methodology

#### Research Duration, Participants' Academic Level, and Sample Size

The most common study samples were small ones with participants range from 11 to 30 (*n* = 11) and medium samples with range between 61 and 90 (*n* = 8) participants. Research durations were mostly between 3 and 6 months (*n* = 10). Small sample size was acknowledged as a limitation in some studies (Hirotani and Fujii, 2019; Zou and Xie, 2019). The possible reason for this is that most of the studies were based on small classroom settings. In the reviewed studies, the most common academic level of participants was undergraduate level. There were 12 studies that did not specify research duration. Regarding this finding, there is a lack of attention in previous retrospective studies (Guan, 2014; Duman et al., 2015; Persson and Nouri, 2018).

#### Data Collection

Most of the studies collected both quantitative and qualitative data, which can help researchers to draw conclusions from different perspectives. Quantitative data included tests, scales, and rubrics; qualitative data included student's work, open-ended questions, student feedback, interviews, student chat transcripts, student reflections, teacher journals, and observations. One of the most common forms of quantitative data collection is a test (*n* = 15), involving student language tests (tests of English speaking and listening) and tests of 21st century skills (critical thinking and creative thinking). The most common method of qualitative data collection was interview (*n* = 13), where the researcher usually designed an interview outline and then asked learners questions to understand their learning experiences, attitudes, motivations, and challenges in the learning process. In addition, researchers have extensively used questionnaires (*n* = 17), including both closed-ended and open-ended questions, to collect both quantitative and qualitative data. For example, the researchers used questionnaires to investigate learners' perceptions of technology-supported language learning, including effectiveness, usefulness, and students' perceptions of developing intercultural communicative competence and language skills through online discussions (Jung et al., 2019).

Based on the above findings, the recommendations of the present study for researchers and teachers are as follow. First, researchers could consider studies with longer time spans and collect data from bigger number of participants to investigate students' development over time and have generalizable conclusions. Second, researchers can collect multiple types of data, focus on students' learning processes and outcomes, and then interpret findings from different perspectives.

#### Research Design

There are a variety of research designs for reviewed studies on technology-supported language learning and 21st century skills. The most common are quasi-experimental studies. Such studies are characterized by using pre- and post-tests to measure changes in participants' language skills, 21st century skills and other learning outcomes and attitudes before and after participation in learning activities. In quasi-experimental studies, participants are not randomly assigned to an experimental or control group (Persson and Nouri, 2018; Huang, 2021). These findings are consistent with other reviews on technology-supported language learning (Persson and Nouri, 2018). The present study suggests that educators and researchers can use the three research methods mentioned above to validate their studies in future.

### Findings

#### Positive Learning Experiences

In this section, the study discusses findings from reviewed studies and recommendations for educators and researchers. In reviewed studies, in addition to finding that technology-supported learning activities promoted learners' language skills and 21st century skills, researchers also found that these technologies led to positive learning experiences, which resulted in better learning outcomes. For example, learning through multimedia textbooks, collaborative blog-based writing activities, smartphone-based video filming activities and language learning projects based on intercultural exchange all increased students' motivation (Amir et al., 2011; García-Sánchez and Burbules, 2016; Sevy-Biloon and Chroman, 2019; Aristizábal-Jiménez, 2020; Huang, 2021). For example, Hosseinpour et al. (2019) noted that through collaborative writing activities, learners' motivation and self-confidence levels were increased. Mirza (2020) argued that through digital storytelling-based learning activities, students gained more confidence. Researchers have also looked at the different learning performance of students due to individual differences in abilities or their characteristics. Yang et al. (2014) found that in terms of writing, significant differences were found between “basic” and “low-intermediate” learners as a result of the difference in ability. Yalçin and Öztürk (2019) found that girls had a more negative attitude toward technology than boys.

#### Challenges Faced by Students

While many studies pointed to positive student attitudes toward technology-supported learning activities (Arnó-Macià and Rueda-Ramos, 2011; Girgin and Cabaroglu, 2021), several studies highlighted challenges that students faced when using technology for learning. Challenges from technology, with some learners finding it difficult to use in learning activities or being confused about the layout of mobile applications were mentioned. Students also noted problems with device incompatibility and poor network quality and speed when using technology. Self-competence challenges, with learners noting that learning tasks were difficult for them, for example, insufficient time to complete learning tasks, lack of research skills, or language skills needed to complete tasks, were reported. Difficulties in finding an interesting topic and choosing the right tools to create their work were also reported in reviewed studies. Challenges of collaborating with others, with some learners noting that they encounter uncoordinated teamwork, uneven distribution of work and unequal student contributions in collaborative tasks, were mentioned by scholars. Self-attitudes, as noted by learners who felt anxious about video chatting when they were communicating remotely, as well as fear of having their writing errors discovered by their partners when communicating in text, were reported in reviewed studies.

Based on the above findings, the present study recommends to educators and researchers, in addition to focusing on the impact of technology-supported learning activities on learners' language skills and 21st century skills, it is also important to focus on students' perceptions of technology, motivation, engagement, and confidence. This is because positive learning experiences can lead to better learning outcomes (Sevy-Biloon and Chroman, 2019; An et al., 2021). Regarding the technological challenges that students encounter in the learning process, it is recommended that they be addressed through advance trainings and through providing students with appropriate technological services during learning activities. Self-competence challenges can be addressed by designing collaborative tasks in which students with higher levels of competence can help students with lower levels of competence to complete the task. Regarding the challenges in collaborative activities, it is recommended that teachers and researchers design learning activities with clear rules for collaborative division of labor and rules regarding how learning performance of every learner will be evaluated. With regard to alleviating negative student attitudes, it is recommended that teachers design diverse teaching strategies and scaffolds to give students assistance during learning activities.

## Conclusion

This study reviewed articles on technology-supported language learning and 21st century skills published from 2011 to 2022 (February) in terms of (a) research focus; (b) theoretical foundations; (c) technology; (d) learning activities; (e) methodology and (f) findings. The results indicate that research on technology-supported language learning and 21st century skills have shown an upward trend in the overall research in the covered time period, with most of the research focusing on English and the majority of participants in these studies majored in education.

Secondly, in terms of research focus, most of the researchers focused on learners' speaking skills (27.40%), followed by writing (26.03%) and vocabulary skills (17.81%). In terms of 21st century skills, most researchers focused on communication (20.83%), collaboration (20.83%), critical thinking (13.89%), and social and cross-cultural interaction skills (13.89%). In terms of theoretical foundations, social constructivist learning theory was most often adopted by researchers. In terms of technology, tools that support learners' creativity and socialization are often utilized by researchers, e.g., Facebook or Google Docs. In terms of learning activities, researchers have designed the following five types of learning activities to support learners' language learning and 21st century skills: (1) collaborative task-based language learning activities; (2) language learning activities based on online communication; (3) creative work-based language learning activities (4) adaptive language learning activities based on learning platforms; and (5) language learning activities based on multimedia learning materials. The results of reviewed studies indicate that these learning activities supported by technology are effective in promoting the development of learners' different language skills and 21st century skills. Finally, in terms of methodology, most of the studies had a sample of 11–30, the most common study period was 3–6 months, the data collection method often used by researchers was questionnaires, the most common method to collect quantitative data was tests, and the most common method to collect qualitative data was interviews.

In contrast to traditional paper and pencil-based learning, technologies used by researchers in reviewed studies allowed learners to improve language learning outcomes and 21st century skills through individual and collaborative learning activities. Some reported advantages are learning with technologies without the constraints of time and space, technologies enable personalized learning, technologies create authentic learning environments that provides adaptive learning content, helps create multimedia content actively, allows social interaction such as sharing, giving or receiving feedback, and reflecting on learning more efficiently.

Based on the above findings, recommendations for researchers and educators in this study include: (1) In terms of language skills, in addition to focusing on output skills, input skills (reading, listening) also deserve attention from researchers. In terms of 21st century skills, learners' problem-solving skills and career and life skills also need more attention from researchers in the future; (2) Advanced technology training for learners to familiarize them with technology and its effective usage as well as teachers need to check in advance for possible technology problems, such as network problems. These suggestions can help teachers address the technological barriers that learners encounter in the learning process; (3) The use of various theoretical approaches, such as instructional design-related theories and language learning-related theories, is important for the rational design of instructional activities that promote learners' language and 21st century skills; (4) Researchers and educators can follow the general model of conducting the five types of instructional activities summarized above to design instructional activities. In addition, it is recommended that researchers and educators use variety of technologies and design different instructional activities to promote learners' language and 21st century skills. It is also important to be aware of the challenges that students may encounter in terms of technology, learning activity tasks, peer collaboration and self-attitudes when implementing learning activities; (5) Teachers and educators could involve more participants and consider longer time spans in future studies to focus on more learners' development and to collect diverse quantitative and qualitative data to explain students' learning processes and outcomes.

There are few limitations to this study. Articles reviewed in this study were sourced from PRIMO and Web of Science databases, and some conference papers, books and dissertations were excluded. For this reason, this study reviewed smaller number of articles. Future studies may consider this limitation and address it by including more relevant sources.

## Data Availability Statement

The raw data supporting the conclusions of this article will be made available by the authors, without undue reservation.

## Author Contributions

RS and XW contributed to the conception, designed the work, collected the data, analyzed, and interpreted data. XW drafted the work and RS substantively revised it. RS was responsible for correspondence. All authors approved the submitted version and agreed both to be personally accountable for the author's own contributions and to the accuracy the work.

## Conflict of Interest

The authors declare that the research was conducted in the absence of any commercial or financial relationships that could be construed as a potential conflict of interest.

## Publisher's Note

All claims expressed in this article are solely those of the authors and do not necessarily represent those of their affiliated organizations, or those of the publisher, the editors and the reviewers. Any product that may be evaluated in this article, or claim that may be made by its manufacturer, is not guaranteed or endorsed by the publisher.
