# Supplementary material for: A Review of Research on Technology-Supported Language Learning and 21st Century Skills
Source: Front Psychol. 2022 Jul 7;13:897689. doi: 10.3389/fpsyg.2022.897689 (PMC9302567; doi:10.3389/fpsyg.2022.897689)
Supplement: Supplementary file 1 [file Table_1.docx]

# Appendices

Appendix 1 Language skills and 21st century skills

| Reference | Language skills | 21st century skills |
| --- | --- | --- |
| Amir et al. (2011) | Writing | Collaboration |
| Arno-Macia & Rueda-Ramos (2011) | Listening  Speaking | Critical thinking |
| Calogerakou & Vlachos (2011) | Writing | Social & cross-cultural interaction  Media literacy |
| Yang et al. (2013) | Listening  Speaking | Critical thinking |
| Thang et al. (2014) | Speaking  Writing | Communication  Collaboration  Creativity and innovation  ICT Literacy |
| Chen & Yang (2014) | Writing  Vocabulary | Communication  Collaboration  Social & cross-cultural interaction |
| Yang et al. (2014) | Reading  Writing | Critical thinking |
| Lewis & Schneider (2015) | Speaking  Grammar | Communication  Social & cross-cultural interaction |
| García-Sánchez & Burbules (2016) | Speaking  Vocabulary | Communication  Collaboration  Problem solving  Digital literacy |
| Chen & Yang (2016) | Speaking  Reading  Writing  Vocabulary | Social & cross-cultural interaction |
| Sevilla-Pavón & Nicolaou (2017) | Listening  Speaking  Reading  Writing  Vocabulary | Communication  Collaboration  Creativity and innovation  Critical thinking  Problem solving  Digital literacy  Social & cross-cultural interaction |
| Lai (2017) | Vocabulary  Grammar | Communication  Collaborative |
| Tseng (2017) | Listening | Social & cross-cultural interaction |
| Özdemir (2017) | Listening  Writing | Social & cross-cultural interaction |
| Srebnaja & Stavicka (2018) | Speaking  Writing  Grammar | Communication  Collaboration  Creativity and innovation  Digital literacy |
| Mohamadi Zenouzagh（2018） | Writing | Collaboration |
| Kulsiri (2018) | Speaking  Reading  Writing  Vocabulary | Collaboration  Creativity and innovation  Problem-solving |
| Zou (2019) | Writing | Collaboration  Critical thinking |
| Yalçin & Öztürk (2019) | Writing | Communication  Collaboration  Creativity and innovation |
| Hosseinpour（2019) | Writing | Collaboration |
| Sevy-Biloon & Chroman (2019) | Speaking | Communication  Social & cross-cultural interaction |
| Valdebenito & Chen (2019) | Listening  Speaking  Writing  Vocabulary  Grammar | Communication  Collaboration  Critical thinking  Digital literacy |
| Jung et al. (2019) | Speaking  Vocabulary  Grammar | Social & cross-cultural interaction |
| Hirotani & Fujii (2019) | Speaking  Grammar | Communication  Social & cross-cultural interaction |
| Huh & Lee (2019) | Speaking  Writing | Creativity and innovation |
| Aristizábal-Jiménez (2020) | Vocabulary  Grammar | Critical thinking |
| Chiang (2020) | Writing | Digital literacy |
| Yang et al. (2020) | Speaking | Creativity and innovation |
| Mirza (2020) | Speaking | Communication |
| Nikitova et al. (2020) | Speaking  Writing  Vocabulary  Grammar | Communication  Collaboration  Critical thinking  Problem solving |
| Huang (2021) | Speaking | Communication  Digital literacy |
| Jamali & Krish (2021) | Speaking  Vocabulary  Grammar | Critical thinking  Digital literacy |
| Girgin & Cabaroğlu (2021) | Listening  Speaking  Reading  Writing  Vocabulary  Grammar | Communication  Collaboration  Critical thinking  Creativity and Innovation |
| Chen et al. (2021) | Vocabulary | Problem solving |

Appendix 2 Theoretical foundation

| Category | Theory | Num | Reference |
| --- | --- | --- | --- |
| Theories related to language learning | Sociocultural theory | 2 | Kulsiri (2018); Mohamadi Zenouzagh (2018) |
|  | Content based instruction | 2 | Tseng (2017); Yang et al. (2013) |
|  | Task based approach to language teaching | 2 | García-Sánchez & Burbules (2016); Lewis & Schneider (2015) |
|  | Context and culture in language teaching | 1 | Calogerakou & Vlachos (2011) |
|  | The input-process-output | 1 | Lai (2017) |
| Theories related to instructional design | Social constructivism | 9 | Girgin & Cabaroğlu (2021); Huang (2021); Jamali & Krish (2021); Lai (2017); Mohamadi Zenouzagh (2018); Sevilla-Pavon & Nicolaou (2017); Yang et al. (2013); Yang et al. (2014); Yang et al. (2020) |
|  | Project-based learning | 2 | Aristizábal-Jiménez (2020); Sevilla-Pavon & Nicolaou, (2017) |
|  | Just-in-time-teaching | 1 | Zou (2019) |
|  | Peer instruction | 1 | Zou (2019) |
|  | Individualized instruction | 1 | Yang et al. (2013) |
|  | Adaptive instruction | 1 | Yang et al. (2014) |
|  | Scaffolding learning theory | 1 | Valdebenito & Chen (2019) |
|  | Problem based learning | 1 | Chen et al.（2021） |
| Theories related to measuring learning outcomes | Byram’s intercultural competence model | 3 | Chen & Yang (2014); Chen & Yang (2016); Özdemir (2017) |
|  | Developmental model of intercultural sensitivity | 1 | Hirotani & Fujii (2019) |
|  | Keller’ ARCS motivational model | 1 | Chen & Yang (2016) |

Appendix 3 Technologies

| Category | Total | Technology | Num | Reference |
| --- | --- | --- | --- | --- |
| Creative  tools | 19 | Windows Movie Maker | 1 | Kulsiri (2018) |
|  |  | iMovie | 1 | Sevilla-Pavón & Nicolaou (2017) |
|  |  | Home Styler | 1 | Lai (2017) |
|  |  | Thing Link | 1 | Lai (2017) |
|  |  | Photo Story 3 | 1 | Thang et al. (2014) |
|  |  | Adobe Spark | 1 | Valdebenito & Chen (2019) |
|  |  |  |  |  |
|  |  | Google My Maps | 1 | Valdebenito & Chen (2019) |
|  |  | Inspiration | 1 | Sevilla-Pavón & Nicolaou (2017) |
|  |  | Audacity | 1 | Yang et al.（2020) |
|  |  | Story Bird | 2 | Chiang (2020), Girgin & Cabaroğlu (2021) |
|  |  | Smartphone camera | 1 | Huang（2021） |
|  |  | Voki | 1 | Girgin & Cabaroğlu (2021) |
|  |  | Go Animate | 1 | Girgin & Cabaroğlu (2021) |
|  |  | Animoto | 1 | Girgin & Cabaroğlu (2021) |
|  |  | Powtoon | 1 | Girgin & Cabaroğlu (2021) |
|  |  | Canva | 1 | Girgin & Cabaroğlu (2021) |
|  |  | Poster MyWall | 1 | Girgin & Cabaroğlu (2021) |
|  |  | Edu Venture | 1 | Chen et al. (2021) |
|  |  |  |  |  |
| Social tools | 20 | Facebook | 4 | Hirotani & Fujii (2019); Sevilla-Pavón & Nicolaou (2017); Sevy-Biloon & Chroman (2019); Özdemir (2017) |
|  |  | Skype | 3 | Chen & Yang (2014); Lewis & Schneider (2015);  Sevy-Biloon & Chroman (2019) |
|  |  | WhatsApp | 2 | Sevilla-Pavón & Nicolaou (2017); Sevy-Biloon & Chroman (2019) |
|  |  | YouTube | 2 | Mirza (2020); Özdemir（2017） |
|  |  | Blog | 3 | Amir et al. (2011); Calogerakou & Vlachos (2011); Thang et al. (2014) |
|  |  | Facetime | 1 | Sevy-Biloon & Chroman (2019) |
|  |  | Live On | 1 | Jung (2019) |
|  |  | ePals | 1 | Chen & Yang (2014) |
|  |  | iEARN | 1 | Chen & Yang (2014) |
|  |  | Google+ Community | 1 | Sevilla-Pavón & Nicolaou (2017) |
|  |  | Google+ Forum | 1 | Sevilla-Pavón & Nicolaou (2017) |
| Collaboration tools | 13 | Google Docs | 4 | Huh & Lee (2019); Sevilla-Pavón & Nicolaou (2017); Valdebenito & Chen (2019); Zou (2019) |
|  |  | Padlet | 3 | Girgin & Cabaroğlu (2021); Lai (2017); Zou (2019) |
|  |  | Google Drive | 2 | Sevilla-Pavón & Nicolaou (2017); Yang et al. (2020) |
|  |  | Wiki | 2 | Chen & Yang (2016); Garcia-Sanchez & Burbules (2016) |
|  |  | E-writing forum | 1 | Mohamadi Zenouzagh (2018) |
|  |  | WordPress | 1 | Valdebenito& Chen (2019) |
| Learning  management system | 9 | Moodle | 4 | Chen & Yang (2016); Garcia-Sanchez & Burbules (2016); Yang et al. (2013); Yang et al. (2014) |
|  |  | Edmodo | 1 | Hosseinpour (2019) |
|  |  | Edpuzzle | 1 | Zou (2019) |
|  |  | Google-classroom | 1 | Yalçın & Öztürk (2019) |
|  |  | Quantum LEAP | 1 | Arnó-Macià & Rueda-Ramos (2011) |
|  |  | WebQuests | 1 | Srebnaja & Stavicka (2018) |
| Classroom interactive tools | 4 | Quizlet | 1 | Girgin & Cabaroğlu (2021) |
|  |  | Quizizz | 1 | Girgin & Cabaroğlu (2021) |
|  |  | Cram | 1 | Girgin & Cabaroğlu (2021) |
|  |  | Kahoot | 1 | Girgin & Cabaroğlu (2021) |
| Multimedia materials | 4 | Multimedia materials | 1 | Tseng (2017) |
|  |  | Audio-visual materials | 1 | Yang et al. (2013) |
|  |  | Film | 1 | Calogerakou & Vlachos (2011) |
|  |  | Multimedia textbooks | 1 | Nikitova et al. (2020) |
| Presentation tools | 2 | PowerPoint | 1 | Mirza (2020); Sevilla-Pavón & Nicolaou (2017) |
|  |  | Prezi | 1 | Sevilla-Pavón & Nicolaou (2017);  Yang et al. (2020) |
| Wearable devices | 1 | Google Cardboard | 1 | Chen et al. (2021) |

Appendix 4 Learning activities

| Categories of learning activities | Num | References |
| --- | --- | --- |
| Collaborative task-based language learning activities | 9 | Amir et al. (2011); Chen et al. (2021); Garcia-Sanchez & Burbules (2016); Girgin & Cabaroğlu (2021); Hosseinpour (2019); Huh & Lee (2019); Lai (2017); Mohamadi Zenouzagh (2018); Valdebenito & Chen (2019) |
| Language learning activities based on online communication | 9 | Calogerakou & Vlachos (2011); Chen & Yang (2014); Chen & Yang (2016); Hirotani & Fujii (2019); Jamali & Krish (2021); Jung (2019); Lewis & Schneider(2015); Özdemir(2017); Sevy-Biloon & Chroman (2019) |
| Creative work-based language learning activities | 8 | Chiang (2020); Huang (2021); Kulsiri (2018); Mirza (2020); Sevilla-Pavón & Nicolaou (2017); Thang et al. (2014); Yalcin (2019); Yang et al. (2020) |
| Adaptive language learning activities based on learning platforms | 4 | Arnó-Macià & Rueda-Ramos (2011); Srebnaja & Stavicka (2018); Yang et al. (2013); Yang et al. (2014) |
| Language learning activities based on learning multimedia materials | 4 | Jiménez (2020); Nikitova et al. (2020); Tseng (2017); Zou (2019) |

Appendix 5 Data collection

| Reference | Data collection |
| --- | --- |
| Arnó-Macià & Rueda-Ramos (2011) | student’s work (blog), open-ended questions, questionnaire |
| Amir et al. (2011) | student’s feedback |
| Calogerakou & Vlachos (2011) | student’s work, questionnaire |
| Yang et al. (2013) | scale (critical thinking skills and critical thinking disposition), English listening and speaking skill test |
| Chen & Yang (2014) | interview, questionnaire |
| Yang et al. (2014) | student discussion notes, TOEIC test, Torrance tests of creative thinking |
| Thang et al. (2014) | interview, questionnaire |
| Lewis & Schneider (2015) | open-ended questions |
| Chen & Yang (2016) | students’ work (students’ written work), student discussion notes (the discussion record from the Moodle course and audio recordings of the group discussions), questionnaire |
| Garcia-Sanchez & Burbules (2016) | reflections |
| Lai (2017) | student’s feedback |
| Özdemir (2017) | interview, student’s work (essay)  scale (measuring online discussion interactions) |
| Sevilla-Pavon & Nicolaou (2017) | questionnaire |
| Tseng (2017) | student discussion notes (students' online conversations) |
| Kulsiri (2018) | interview, questionnaire |
| Srebnaja & Stavicka (2018) | reflections  questionnaire |
| Mohamadi Zenouzagh (2018) | Interview, student’s work, teacher journal, questionnaire, test (language proficiency test), textuality measurement rubric |
| Hirotani & Fujii (2019) | observation, Student discussion notes (forum comments) |
| Hosseinpour（2019) | students’ work (mini video draft), student’s feedback |
| Huh & Lee (2019) | students’ work (mind map), test (Torrance Test of Creative Thinking, English language skills), questionnaire |
| Jung et al. (2019) | Student discussion notes, questionnaire |
| Sevy-Biloon & Chroman (2019) | students’ work (blog writing), questionnaire, oral test, student discussion notes |
| Valdebenito & Chen (2019) | interview, students’ work (essay) |
| Yalcin (2019) | students’ work (essays), reflections, California Critical Thinking Skills Test, The Test of English for International Communication, Holistic Critical Thinking Scoring Rubric |
| Zou (2019) | student’s work (essay), interview, Torrance Tests of Creative Thinking |
| Aristizábal-Jiménez (2020) | observation, interview, diagnostic writing test, questionnaire  students’ work, teacher’s journal |
| Chiang (2020) | interview, open-ended questions, scale (digital literacy, self-efficacy scale for using English as an international language) |
| Mirza (2020) | open-ended questions |
| Nikitova et al. (2020) | questionnaire (learning preference，learning needs，course satisfaction), English Language Learning Achievement Test |
| Yang et al. (2020) | test (GEPT subtest on speaking, Torrance Tests of Creative Thinking) |
| Chen et al. (2021） | interview，student‘s work, questionnaire (English Learning Motivation questionnaire), English Language Learning Achievement Test |
| Girgin & Cabaroğlu (2021) | questionnaire, observation, teacher journal, interview |
| Huang (2021) | interview, reflections, questionnaire, oral performance test |
| Jamali & Krish (2021) | open-ended questions, interview |

Appendix 6 Study design

| Research design | Num | Reference |
| --- | --- | --- |
| Quasi-experiment  (A pretest–posttest design) | 14 | Chen et al. (2021); Chiang (2020); Huang (2021); Hirotani & Fujii (2019); Hosseinpour (2019); Huh & Lee (2019); Mohamadi Zenouzagh (2018); Nikitova et al. (2020); Özdemir (2017); Thang et al. (2014); Zou (2019); Yang et al. (2020); Yang et al. (2014); Yang et al. (2013) |
| Case study | 12 | Amir et al. (2011); Calogerakou & Vlachos (2011); Chen & Yang (2016); García-Sánchez & Burbules (2016); Jamali & Krish (2021); Jung et al. (2019); Mirza (2020); Lai (2017); Sevilla-Pavón & Nicolaou (2017); Sevy-Biloon & Chroman (2019); Valdebenito & Chen (2019); Yalçin & Öztürk (2019) |
| Action study | 8 | Aristizábal-Jiménez (2020); Arno-Macia & Rueda-Ramos (2011); Chen & Yang (2014); Girgin & Cabaroğlu (2021); Kulsiri (2018); Lewis & Schneider (2015); Srebnaja & Stavicka (2018); Tseng (2017) |

Appendix 7 Findings

| Reference | Results | Challenges for participate |
| --- | --- | --- |
| Amir et al. (2011) | The adoption of using blogs as instructional technology facilitate learners' collaborative writing processes and interactions, in which they can participate actively while learning. | Not specified |
| Arno-Macia & Rueda-Ramos (2011) | The online learning environment promotes their listening and speaking skills and critical thinking skills, while enhancing learners' motivation. | Not specified |
| Calogerakou & Vlachos (2011) | Cultural programs based on film and blog writing promote learners' writing, collaboration and cross-cultural interaction skills and enhance learner motivation | Not specified |
| Yang et al. (2013) | Online critical thinking-integrated individualized English instruction had a positive impact on learners' English listening and speaking and critical thinking skill. | Not specified |
| Thang et al. (2014) | The use of digital storytelling in English for Academic Purpose (EAP) course promotes students' speaking, writing, communication, creativity, writing and ICT skills. | Not specified |
| Chen & Yang (2014) | Students made significant progress in cross-cultural understanding, critical cultural thinking and communication skills and writing, vocabulary skills. | The challenge of self-competence: Student lack of research skills, did not know how to formulate a valid research question and Summarizing information. Student lack of language use skills, inadequate writing strategies and in understanding their partners' English questions and limited vocabulary. |
| Yang et al. (2014) | Promoted students' reading and writing skills, as well as critical thinking skills. In terms of reading, “High-advanced” learners, unsurprisingly, noted less improvement than “low-advanced” learners. In terms of writing, significant differences were found between basic and “low-intermediate” learners as well as “high-intermediate” and “low-advanced” learners. | Not specified |
| Lewis & Schneider (2015) | The Virtual Language Exchange experience proved positive and beneficial to the students. Students develop speaking, grammar and communication skills in the process of virtual language communication. | Not specified |
| García-Sánchez & Burbules (2016) | Students' skills (such as problem solving, collaboration, listening and speaking) improved after they completed online collaborative tasks. | The challenge of self-competence: learners find the learning task challenging for them. |
| Chen & Yang (2016) | This project improved student’s vocabulary, writing skills, and collaboration, cross-cultural interaction skill. The students' experiences revealed that the multimodal design lacked transformation between tasks and had no active effect on lowering anxiety. | The challenge of self-competence: low vocabulary knowledge; Unfamiliar accent, grammar. |
| Sevilla-Pavón & Nicolaou (2017) | Through the DST project, students think their basic linguistic skills (reading, writing, listening, speaking, vocabulary) and research skills, digital skills, as well creativity, innovation, critical thinking, problem solving, communication and collaboration skills were developed. | The challenge of self-competence: limited time available for completing the task.  The challenge of collaborating with others: The coordination of the team in local and telecollaboration respects. Work was not allocated in equal terms and the students’ contributions were not balanced. |
| Lai (2017) | This project improved students' vocabulary and grammar skills. The online tools also facilitate communication, sharing and collaboration between teachers and students. | The challenge from technology: The software is a bit of an obstacle about ThingLink and Homestyler. |
| Tseng (2017) | Student view video clips (i.e., Ted. Talks, Voice Tubes) and movies were also reported to be interesting and helpful in enhancing their listening skills. They learned different beliefs, attitudes, and communication styles associated with different cultures from lectures, their own research projects, and their peer report, this process promotes students' intercultural skills. | Not specified |
| Özdemir  (2017) | The intercultural instruction had a significantly positive effect on learners’ intercultural communicative effectiveness (ICE). The discussion on Facebook group had significantly higher ICE scores than the in-class discussion group. The students especially highlighted the improvements in reading, writing, and listening skills. | Attitude: Students feel anxious when they use Facebook. They are afraid of making mistakes in their writing and being seen by others. |
| Srebnaja & Stavicka (2018) | Through the WebQuest project, Students think their ability about interact, collaborate, and communicate with others, digital literacy skills, information literacy skills, innovation, writing skill are promoted, and they made fewer grammar, spelling mistakes. | The challenge of collaborating with others: Students said the team cooperation is not smooth. |
| Mohamadi Zenouzagh (2018) | Students' writing skills and collaboration skills are developed through collaborative writing activities supported by technology. | The challenge from technology: Internet quality and speed. The challenge of collaborating with others: Their partners’ not doing their responsibilities on time and making. Their partners’ delays in group work disrespecting others’ ideas. |
| Kulsiri (2018) | Through Student-Produced Video project, students think that speaking, reading and writing skills have been improved, but listening skills have not been improved, and reading skills and vocabulary have been improved more than other skills. Students feel more willing to cooperate with their peers in this process, and their problem-solving, creative ability has also been improved. | Not specified |
| Zou (2019) | The experimental group had higher writing skills, as well as critical thinking skills and collaboration skills than the control group. | Not specified |
| Yalçin & Öztürk (2019) | Students' writing skills improve as a result of participating in learning activities, while collaboration, communication and creativity skills are promoted. Both male and female students preferred individual study rather than the team working. Girl’s attitudes toward digital tools in literature more negative than boy’s. | The challenge of self-competence: students think that digital storytelling takes much time. |
| Hosseinpour (2019) | Most learners, especially Edmodo high users, had a positive perception of the collaborative writing as it could increase their level of motivation and self-confidence. Blended Learning improved academic writing proficiency of Iranian intermediate EFL learners through Edmodo mobile application. However, this technique did not promote all five aspects of the writing skill in the same manner. | The challenge of self-competence: writing time is short. The challenge from technology: students are confused about the layout of mobile applications. device incompatibility and connection problems. |
| Sevy-Biloon & Chroman (2019) | Student feel more comfortable speaking in English and have increased in oral communication level. And this project promoted students' cross-culture interaction skills. After participate in the video chat, students had positive experiences, increased motivation and improved communication skills. | Not specified |
| Valdebenito & Chen (2019) | Through the Food Project, improved students' language skills (writing, reading, listening, vocabulary, grammar, speaking), and digital literacy skills, cross-cultural skills, communication and critical thinking skills. | Not specified |
| Jung et al. (2019) | Video chat is helpful to improve students' speaking, vocabulary and grammar skills. In this process, students eliminate cultural misunderstanding, gain cultural knowledge and improve their intercultural communication ability. | Not specified |
| Hirotani & Fujii (2019) | Through this project, students believe that their speaking skills have been improved, they can prepare to use grammar to express. Their cross-cultural skills and communication skills have also been improved. | Not specified |
| Huh & Lee (2019) | Students' speaking and writing skills and creativity improved through this project. | Not specified |
| Aristizábal-Jiménez (2020) | The use of multimedia textbooks gives students a greater desire to work with computer-assisted assignments, increases motivation and engagement in learning, and promotes vocabulary and grammar, and critical thinking skills. | The challenge of self-competence: Feelings of difficulty in learning content. |
| Chiang (2020) | There were significant differences in the overall digital literacy between the experimental group and the control group, the participants made the most substantial gain in reproduction/visual literacy while the least in information literacy. There were no significant differences in the self-efficacy between the experimental group and the control group, but the open-ended survey showed that most participants thought their writing self-efficacy had improved after a year of writing training. | The challenge of self-competence: Students struggled with the learning tasks of choosing appropriate artwork to describe and selecting appropriate images to support the writing. |
| Yang et al. (2020) | DST-based instruction had a positive effect on the students’ English speaking and creative thinking. | Not specified |
| Mirza (2020) | Many participants also reported having gained more self-confidence and improved their speaking, organizational, technical and research skills, as well as their communication skill. | The challenge of self-competence: difficult to find an interesting topic and difficult to create PPT, find the right video application and inserting audio. |
| Nikitova et al. (2020) | Students' speaking, writing, grammar and vocabulary skills, as well as collaboration, communication, problem solving and critical thinking skills, are enhanced through the study of multimedia materials and the completion of learning tasks. | The challenge of self-competence: Feelings of difficulty in learning content. |
| Huang (2021) | Integrating collaborative vlog projects in EFL classrooms can stimulate students’ speaking performance. Student-made collaborative vlogs can help students develop digital literacy skill. Smartphone-based vlog projects can increase learners’ engagement and enhance their group collaboration skills. | The challenge of self-competence: the difficulty of shooting the video, as well as the time-consuming post-editing. |
| Jamali & Krish (2021) | Online discussion forums promote students' grammar, vocabulary, and speaking skills, as well as critical thinking and digital literacy skills | The challenge from technology: Quizlet is a difficult tool to use |
| Girgin & Cabaroğlu (2021) | Participants had positive attitudes toward flipped classroom learning that integrated Web 2.0 tools, enhanced motivation and engagement, and improved students' vocabulary, grammar, reading, listening, speaking, and writing skills, and 4C skills (i.e., critical thinking, creative thinking, communication, and collaboration). | Not specified |
| Chen et al. (2021) | The project integrating virtual reality technology with the PBL environment promoted students' vocabulary skills and problem solving skill, as well as increased their motivation. | Not specified |

Appendix 8 The most commonly used technologies to facilitate 21st century skills

| 21st century skills | Tools (categories) | Reference |
| --- | --- | --- |
| Communication | Facebook (Social tools) | García-Sánchez & Burbules (2016)  Hirotani & Fujii (2019)  Sevy-Biloon & Chroman (2019) |
| Collaboration | Google Docs (Collaboration tools) | Sevilla-Pavón & Nicolaou (2017)  Valdebenito & Chen (2019)  Zou (2019) |
| Critical thinking | Google Docs (Collaboration tools) | Sevilla-Pavón & Nicolaou (2017)  Valdebenito & Chen (2019) |
|  | Moodle (Learning management system) | Yang et al. (2013); Yang et al. (2014) |
|  | Padlet (Collaboration tools) | Girgin & Cabaroğlu (2021); Zou (2019) |
| Creativity and innovation | Google Docs (Collaboration tools) | Huh & Lee (2019)  Sevilla-Pavón & Nicolaou (2017) |
|  | Prezi (Presentation tools) | Sevilla-Pavón & Nicolaou (2017)  Yang et al. (2020) |
| Problem solving | Edu Venture (Creative tools) | Chen et al.（2021） |
|  | i Movie (Creative tools) | Sevilla-Pavón & Nicolaou (2017) |
|  | Inspiration (Creative tools) | Sevilla-Pavón & Nicolaou (2017) |
| Social & cross-cultural interaction | Facebook (Social tools) | Hirotani & Fujii (2019)  Sevilla-Pavón & Nicolaou (2017)  Sevy-Biloon & Chroman (2019)  Özdemir (2017) |
| Digital literacy | Google Docs (Collaboration tools) | Sevilla-Pavón & Nicolaou (2017)  Valdebenito & Chen (2019) |
| ICT Literacy | Windows Movie Maker (Creative tools) | Kulsiri (2018) |
|  | Photo Story3 (Creative tools) | Thang et al. (2014) |
|  | Blog (Social tools) | Thang et al. (2014) |
| Media literacy | Film (Multimedia materials) | Calogerakou & Vlachos (2011) |
|  | Blog (Social tools) | Calogerakou & Vlachos (2011) |
